# Cellular and physiological circadian mechanisms drive diurnal cell proliferation and expansion of white adipose tissue

**DOI:** 10.1038/s41467-021-23770-0

**Published:** 2021-06-09

**Authors:** Aleix Ribas-Latre, Rafael Bravo Santos, Baharan Fekry, Yomna M. Tamim, Samay Shivshankar, Alaa M. T. Mohamed, Corrine Baumgartner, Christopher Kwok, Claudia Gebhardt, Angielyn Rivera, Zhanguo Gao, Kai Sun, John T. Heiker, Brad E. Snyder, Mikhail G. Kolonin, Kristin L. Eckel-Mahan

**Affiliations:** 1 Institute of Molecular Medicine, McGovern Medical School at the University of Texas Health Science Center, Houston, TX 77030 USA; 2 Helmholtz Institute for Metabolic, Obesity and Vascular Research (HI-MAG) of the Helmholtz Zentrum München at the University of Leipzig and University Hospital Leipzig, 04103 Leipzig, Germany; 3 Faculty of Medicine, Ain Shams University, Cairo, 1181 Egypt; 4 Memorial Hermann Texas Medical Center, Houston, TX 77030 USA; 5 Department of Integrative Biology and Pharmacology, McGovern Medical School at the University of Texas Health Science Center, Houston, TX 77030 USA

**Keywords:** Fat metabolism, Feeding behaviour, Obesity, Metabolic diseases, Type 2 diabetes

## Abstract

Hyperplastic expansion of white adipose tissue (WAT) relies in part on the proliferation of adipocyte precursor cells residing in the stromal vascular cell fraction (SVF) of WAT. This study reveals a circadian clock- and feeding-induced diurnal pattern of cell proliferation in the SVF of visceral and subcutaneous WAT in vivo, with higher proliferation of visceral adipocyte progenitor cells subsequent to feeding in lean mice. Fasting or loss of rhythmic feeding eliminates this diurnal proliferation, while high fat feeding or genetic disruption of the molecular circadian clock modifies the temporal expression of proliferation genes and impinges on diurnal SVF proliferation in eWAT. Surprisingly, high fat diet reversal, sufficient to reverse elevated SVF proliferation in eWAT, was insufficient in restoring diurnal patterns of SVF proliferation, suggesting that high fat diet induces a sustained disruption of the adipose circadian clock. In conclusion, the circadian clock and feeding simultaneously impart dynamic, regulatory control of adipocyte progenitor proliferation, which may be a critical determinant of adipose tissue expansion and health over time.

## Abstract (web summary)

During the expansion of adipose tissue adipocyte progenitor cells proliferate and undergo adipogenesis. Here, the authors show that adipocyte progenitor cell proliferation in visceral adipose tissue has a diurnal pattern, which is dependent on both energy intake and the circadian clock.

## Introduction

Adipose tissue is a metabolic organ with tremendous plasticity to retract and expand, processes that are driven via the coordination of a wide variety of cells within the adipose tissue^1^. Adipose tissue is comprised of mature adipocytes (MA) and resident stromal vascular cell fraction (SVF), which include adipocyte progenitor cells (APCs) having the ability to proliferate and undergo adipogenesis to form MA. Enlarged adipose tissue can be due to an elevated number of adipocytes comprising the white adipose tissue (WAT) pad (hyperplasia), or an increase in the size of resident adipocytes (hypertrophy), both of which are dependent on dynamic processes and heavily influenced by diet^2^. Recent studies suggest that in previously obese individuals who have lost weight, adipocyte hypertrophy is reduced, while the total cell number remains more or less constant^3^. Thus, proliferation of pre-adipocytes may irrevocably alter WAT, regardless of total mass. While high-fat diet (HFD) feeding induces pre-adipocyte proliferation, the extent to which the temporal aspects of feeding and circadian mechanisms contribute to pre-adipocyte proliferation and adipose tissue mass in vivo is not well understood.

Similar to other peripheral organs studied from a circadian context, adipocytes and adipose tissue are highly rhythmic in rodents and humans alike, with similar phase oscillations for the core clock components in brown adipose tissue (BAT), inguinal (iWAT), and epididymal (eWAT)^4–11^. Prior studies addressing the effects of feeding on WAT have shown that it is tightly regulated by energy intake, with temporal restriction of feeding causing changes in the core clock genes and their targets^9^. A recent study analyzing subcutaneous adipose biopsies from healthy subjects under constant routine conditions revealed over 800 rhythmic transcripts in WAT^4^. While transcripts of genes involved in transcription regulation were heavily represented in the morning-peaking transcript group, evening-peaking transcripts were composed of a significant number of genes involved in redox activity. The importance of the circadian clock in adipose tissue is supported by several circadian-disrupted mouse models^12,13^, including knockouts of or mutations in the core clock transcriptional activators Circadian Locomotor Output Cycles protein Kaput (*Clock*) and Aryl hydrocarbon Receptor Nuclear Translocator Like (*Arntl*, also known as *Bmal1*)^14^. For example, *Clock* mutant mice, which express mutant CLOCK protein in all cells, become more obese and insulin resistant on a HFD compared to wild-type (WT) littermate controls^15^. Loss of BMAL1 globally leads to increased adipogenesis and adipocyte hypertrophy^16^, while adipocyte-specific loss of BMAL1 causes a defective circadian release of polyunsaturated fatty acids, and ultimately increases adiposity coincident with arrhythmic energy intake^8^. Furthermore, adipogenesis is stimulated by the circadian deadenylase, Nocturnin (*Noc*), in a peroxisome proliferator-activated receptor gamma (PPARG)-dependent manner^17^. Also affecting PPARG in fat is the circadian transcriptional repressor Period 2, *Per2*, the loss of which results in enhanced PPARG recruitment to target sites of adipogenic genes^18^. The Period 3 (*Per3*) gene is known to have a particularly high amplitude oscillation in pre-adipocytes, where it too limits adipogenesis by repressing Krüppel-like factor 15 (*Klf15*) expression^19^. CLOCK protein is also thought to act in pre-adipocytes, where it attenuates adipogenesis via the transcriptional regulation of glucocorticoid‐induced leucine zipper (GILZ)^20^. In conclusion, components of the circadian clock regulate adipogenesis and play a role in systemic energy balance.

Complicating the role of the clock in fat is that rhythmicity of peripheral tissues, including adipose tissue, is heavily controlled by energy intake as it pertains to both the timing and quality^6,9,21–26^. For example, HFD can reprogram some peripheral clocks in a reversible manner^6,21,27^. However, prolonged HFD feeding induces a loss of circadian coordination across tissues^6^. In contrast, restricted feeding, even under HFD, can prevent obesity and adiposity, and improve the robustness of circadian rhythms in peripheral tissues^24,25^. However, the effects of nutrient challenge in adipose tissue at the cellular level is poorly understood. In this study, we reveal a pronounced diurnal variance of adipose tissue stromal vascular cell proliferation in vivo. While this proliferation is predominantly due to pre-adipocytes in VAT, CD31-positive cells in SAT also contribute to this diurnal variance. While disrupting rhythmic energy intake is sufficient to abolish this diurnal variation in pre-adipocyte proliferation, so is nutrient challenge in the form of HFD feeding. Though feeding appears to be a key regulator of diurnal proliferation within adipose tissue, data from *Clock*-deficient mice suggests that both feeding and the circadian clock regulate rhythmicity in SVF proliferation. Finally, analysis of pro-proliferative markers in human subcutaneous and visceral WAT in non-obese vs. obese humans suggests that similar regulatory mechanisms of diurnal proliferation may be present in human fat. Collectively, these data suggest that cellular and physiological circadian rhythms may be directly involved in the maintenance of healthy adipocyte progenitor pools in adipose tissue over time, ultimately contributing to both adipose tissue mass, and health in the context of adipose tissue expansion.

## Results

### Proliferation of SVF in WAT shows a diurnal pattern in vivo

**Fig. 1:**
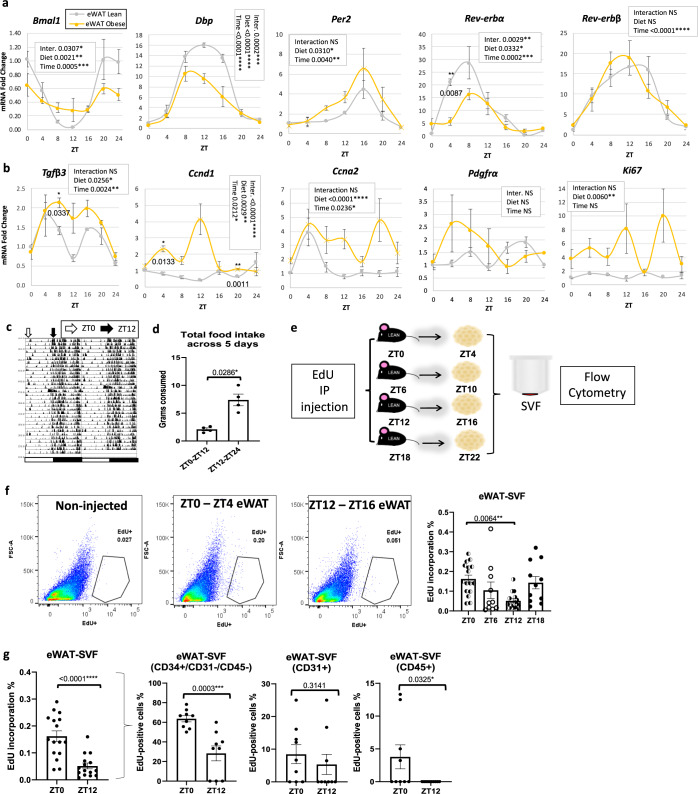
Diurnal variation in the proliferation of stromal vascular cells in vivo. **a, b** RT-PCR analysis reveals expression of circadian (**a**) and proliferation (**b**) genes in the epididymal adipose tissue (eWAT) from lean (gray) and obese (yellow) mice throughout the circadian cycle (*n*  =  4 animals/zeitgeber time, ZT). mRNA levels for lean mice at ZT0 were set to 1 for all genes. **c** Diurnal locomotion of mice measured by infrared sensors. White and black arrows indicate the beginning of the light (ZT0) or the dark (ZT12) phase, respectively. **d** Diurnal food intake of mice fed vivarium chow ad libitum (*n* = 4 animals/ light or dark period). **e** Experimental model: 5-ethynyl-2ʹ-deoxyuridine (EdU) was intraperitoneally (IP) injected to lean mice fed ad libitum control chow at ZT0, ZT6, ZT12, or ZT18. eWAT and inguinal adipose tissue (iWAT) stromal vascular cell fractions (SVF) were isolated from fat pads harvested 4 h following EdU injection. **f** Representative fluorescence activated cell sorting of EdU-positive cells in eWAT-SVF isolated at ZT4 and ZT16, 4 h following a single EdU injection (left panel). Right panel: quantification of fluorescence activated cell sorting of EdU-positive cells from eWAT SVF isolated 4 h following EdU injections at ZT0, ZT6, ZT12, and ZT18 (*n* = 17 animals in ZT0 and ZT12 and *n* = 12 animals in ZT6 and ZT18). **g** Quantification for total EdU^+^ SVF (*n* = 12–17 animals/ZT) isolated 4 h following EdU injections at ZT0 and ZT12 (left panel) and EdU^+^/CD34^+^/CD31^−^/CD45^−^, EdU^+^/CD31^+^, and EdU^+^/CD45^+^ (*n* = 10 animals/ZT) SVF (middle and right panels, respectively). Significance (p < 0.05) determined by two-way ANOVA and Sidak’s post-hoc test in a-b; two-tailed Mann–Whitney U test in d and g (middle and right panels); two-tailed unpaired T-test in g (left panel); and one-way ANOVA [F(3, 48) = 4.407] followed by Tukey’s post-hoc test in f.

To determine how genes that might be involved in adipocyte progenitor proliferation are expressed throughout the 24 h cycle in lean vs. obese mice, iWAT, and eWAT were taken from lean and diet-induced obese (DIO) mice at different time points throughout the diurnal cycle (Figs. 1a, b and S1a, b). Analysis of core clock genes in lean vs. obese mice revealed oscillation of the circadian transcription factor *Bmal1* in both eWAT and iWAT of lean animals (Figs. 1a and S1a). *Bmal1* continued to oscillate in iWAT and eWAT under conditions of DIO, based on JTK_Cycle rhythmicity tests^28^ as well as cosinor analysis (Fig. 1a and Supplementary Dataset 1). D-Box Binding PAR BZIP Transcription Factor (*Dbp*), a target gene of CLOCK:BMAL1, was rhythmic in lean and obese conditions in both WAT depots, though its amplitude was reduced in DIO (Figs. 1a and S1a). *Per2* and Nuclear Receptor Subfamily 1 Group D Members 1 and 2 (*Nr1d1/Rev-erbα* and *Nr1d2/Rev-erb*) were rhythmic in both depots and diet conditions, with the exception of *Rev-erbα*, which lost rhythmicity in iWAT in HFD (Figs. 1a and S1a, and Supplementary Dataset 1). In addition to analyzing clock genes in WAT, we look at the diurnal expression of several genes involved in cell proliferation. Transforming growth factor beta 3 (*Tgfβ3*), a gene recently identified to promote adipocyte progenitor proliferation^3^, was greatly induced under HFD at most of the diurnal time points tested and became rhythmic under HFD in eWAT (Figs. 1b and S1b, and Supplementary Dataset 1). The cell cycle regulator cyclin D1^29^ (*Ccnd1*) was also induced and became rhythmic in eWAT on HFD (Figs. 1b and S1b, and Supplementary Dataset 1). Other markers or drivers of proliferation, including cyclin A2 (*Ccna2*), platelet-derived growth factor receptor A (*Pdgfra*), and marker of proliferation Ki-67 (*Ki67*) were either oscillatory under both feeding conditions or induced and rhythmic under HFD in at least one of the two depots (Figs. 1b and S1b, and Supplementary Dataset 1).

**Fig. 2:**
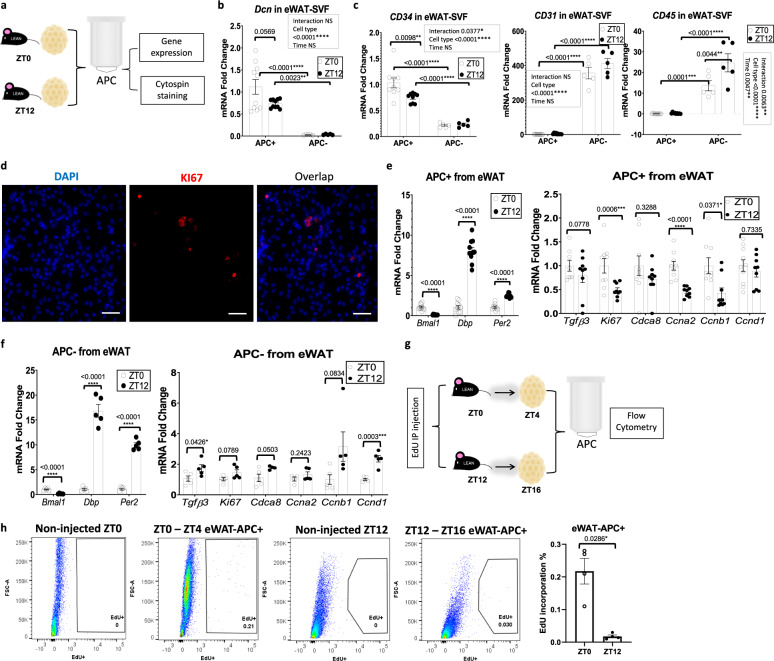
Diurnal proliferation of adipocyte progenitor cells (APCs) in epididymal adipose tissue (eWAT). **a** Experimental model: adipose progenitor cells (APC) within the eWAT and iWAT stromal vascular cells were isolated at ZT0 and ZT12 from lean mice fed ad libitum control chow, for further gene expression and immunostaining analysis. **b**, **c** RT-PCR analysis reveals expression of progenitor (Dcn and CD34), endothelial (CD31), and lymphocyte (CD45) markers in eWAT/APC and eWAT/non-APC cells from lean mice isolated at ZT0 and ZT12 (*n* = 5 animals for APC^−^ and *n* = 10 animals for APC^+^/zeitgeber time, ZT). mRNA levels for lean mice at APC^+^/ZT0 were set to 1 for all genes. **d** Representative staining of KI67 (vs. DAPI) in eWAT-APC (scale bar = 50 µm). Staining performed once from 3 biological replicates. **e**, **f** RT-PCR analysis reveals expression of circadian (left panel) and proliferation genes (right panel) in eWAT/APC (**e**) and eWAT**/**non-APC cells (**f**) from lean mice isolated at ZT0 and ZT12 (*n* = 5 animals for APC^−^ and *n* = 10 animals for APC^+^/ZT). mRNA levels for lean mice at APC^+^/ZT0 were set to 1 for all genes. **g** Experimental model: EdU was administered to lean mice fed ad libitum control chow at ZT0 and ZT12. eWAT and iWAT SVF were isolated from fat pads harvested 4 h following EdU injection and the subsequent adipose APC were isolated for further analysis. **h** Representative fluorescence activated cell sorting of EdU-positive cells in eWAT-APC isolated at ZT4 and ZT16, 4 h following a single EdU injection (left panel). Right panel: Quantification for total EdU^+^ APC (*n* = 5 animals/ZT). For all bar graphs, white and black circles represent ZT0 and ZT12, respectively. Data are represented as mean ± SEM. ^*^p < 0.05, ^**^p < 0.01, ^***^p < 0.001, ^****^p < 0.0001. Significance (*p* < 0.05) determined by two-way ANOVA and Tukey’s post-hoc test in b-c; two-tailed unpaired T-test in e-f; and two-tailed Mann–Whitney U test in h.

While MA undergo limited proliferation^30–33^, adipocyte progenitors are known to divide prior to maturing into MA^32^. To test whether rhythmicity in proliferation genes in adipose tissue may be indicative of diurnal cell proliferation in vivo, lean mice fed chow diet ad libitum were given intraperitoneal injections of 5-ethynyl-2ʹ-deoxyuridine (EdU), which gets incorporated into DNA during the process of cell division. Though a single injection of EdU produced significantly less EdU-positive SVFs than 3 consecutive days of single injections at ZT0 in separate cohorts of lean mice (Fig. S1c), a single injection of EdU at ZT0 (“lights on” in the room) was sufficient to detect SVF proliferation in vivo. To test for rhythmic proliferation of cells in the SVF in vivo, mice fed ad libitum were injected at ZT0, ZT6, ZT12, or ZT18 (ZT0 being the beginning of the dark/active/feeding phase). Because mice generally lose weight in metabolic cages, a separate cohort of lean mice were placed in circadian and metabolic chambers to measure rhythmicity in locomotion and energy intake under the same ad libitum feeding conditions (Fig. 1c, d). Adipose tissues removed from mice 4 h following EdU injection was digested, fractionated, and the SVFs were sorted for EdU^+^ cells (Fig. 1e). Remarkably, SVFs of mice injected with EdU at the end of the feeding phase (ZT0) revealed 2- to 3-fold more EdU^+^ cells compared to mice injected with EdU at the end of the fasting phase (ZT12), depending on the depot, with mid-fasting phase (i.e., ZT6) levels also being considerably lower compared to the feeding phase time points (Figs. 1f and S2a) (*n* = 12–17/condition). To determine the cellular identity of proliferating cells, eWAT and iWAT SVFs were prepared from mice injected with EdU at ZT0 or ZT12 and stained and sorted for the presence of EdU, endothelial marker CD31, hematopoietic marker CD45, and the stem cell marker CD34. CD34^+^/CD31^−^/CD45^−^ cells are generally considered to be adipocyte progenitors, while CD34^−^/CD31^−^/CD45^+^ cells are considered to be hematopoietic^30,34–37^. Adipocyte progenitor proliferation (as defined by CD34^+^/CD31^−^/CD45^−^ cells) were strongly rhythmic in eWAT and iWAT (Figs. 1g and S2b). Though CD45^+^ cells showed a diurnal pattern of proliferation aligned with the pattern of total EdU-positive cells in eWAT, the percent of EdU^+^/CD45^+^ cells was small compared to the EdU^+^/CD34^+^/CD31^−^/CD45^−^ cells. In contrast to eWAT, EdU^+^/CD45^+^ cells in iWAT showed almost no proliferation, diurnal or otherwise, though EdU^+^/CD31^+^ cells also showed diurnal variation in phase with adipocyte progenitors (Fig. S2b). To further validate that APCs specifically display a diurnal pattern of proliferation, eWAT and iWAT was harvested from mice at ZT0 and ZT12, followed by APC isolation to generate pure APCs vs. all remaining cell types (primarily endothelial and immune cells^38^). Isolated APCs were subjected to either RT-PCR analysis to analyze gene expression, or else deposited by a cytocentrifuge onto slides for staining with the cell proliferation marker, Ki67 (Fig. 2a–f). APC vs. non-APC fractions were validated by gene expression of markers of stromal cell origin, including decorin (*Dcn*) and CD34, as well as markers of endothelial and immune origins (CD31 and CD45, respectively) (Figs. 2b, c and S2c for iWAT). Staining of plated APCs from eWAT with a Ki67 antibody revealed active proliferation of APCs (Fig. 2d). To determine whether purified APCs from eWAT expressed the normal diurnal pattern of circadian clock genes, purified APC and non-APC fractions were analyzed for expression of *Bmal1*, *Dbp*, and *Per2* (Fig. 2e, f). As expected, anti-phase rhythms of *Dbp* and *Per2* with *Bmal1* was noted, as was the anticipated expression patterns at the two zeitgeber times (ZT), considering the common phase of expression for these genes across tissues^10^. To test for diurnal changes in genes associated with cell proliferation in purified progenitor cells of eWAT, purified APCs were analyzed for previously tested proliferation markers as well as *Ccnb1* and *Cdca8*, a gene found by single-cell RNA-seq experiments to be highly elevated in a subset of proliferative adipocyte progenitors^39^. In contrast to clock genes, proliferation genes showed a distinct profile in eWAT-APCs, with ZT12 levels generally being lower than ZT0 (Fig. 2e, right panel). Cells comprising the non-APC fraction showed the expected phase of clock gene expression; however, proliferation gene expression did not mirror the pattern seen in APCs (Fig. 2f). To further confirm that APCs undergo diurnal proliferation in SVF, we injected mice with EdU at ZT0 or ZT12, and purified APCs from eWAT and iWAT harvested 4 h following EdU injection (Fig. 2g). APCs from eWAT showed a pronounced diurnal variation in proliferation, with proliferation much higher at the end of the feeding phase (ZT0) compared to ZT12 (Fig. 2h). While EdU^+^/CD34^+^/CD31^−^/CD45^−^ revealed diurnal proliferation in iWAT (Fig. S2a, b), Sca1-purified APCs from iWAT trended towards reduced proliferation at ZT12 compared to ZT0 (Fig. S2c, d), but did not reach significance, suggesting that at least some of the diurnal cell proliferation observed in iWAT (Fig. S2a) may be a result of diurnally proliferating endothelial cells. To summarize, in lean, rhythmic, ad libitum-fed mice, there is a pronounced diurnal variance in proliferation of SVF cells in eWAT and iWAT, which is largely comprised of adipocyte progenitors in eWAT, and also involves endothelial cell proliferation in iWAT.

### High-fat diet disrupts the diurnal balance of APC proliferation

HFD promotes pre-adipocyte proliferation within both eWAT and iWAT^40,41^, though proliferation of adipose tissue macrophages can also occur^42^. Based on the pronounced upregulation of proliferation markers in whole WAT under the context of HFD feeding in mice, we tested whether cells comprising the SVF showed a change in pro-mitogenic gene expression patterns in DIO. To determine whether expression of genes involved in cell proliferation was altered in the context of HFD specifically within SVFs, we took WAT from lean and DIO mice throughout the circadian cycle and isolated SVFs were analyzed for circadian gene expression as well as drivers of proliferation, including *Ki67* and *Pdgfra* expression, Fig. S3a, b. (While Ki67 expression does vary throughout the cell cycle^43^, it is a marker of cell proliferation, peaking during the G2 phase and mitosis^44^.) Though iWAT SVFs from mice fed HFD appeared to be less susceptible to disruption of circadian gene expression throughout the diurnal cycle, mice fed prolonged HFD showed marked changes in *Bmal1* and *Per2* rhythmicity in eWAT SVFs, as well as robust induction of *Ki67* and *Pdgfra* at specific ZTs (Fig. S3b and Supplementary Dataset 1).

**Fig. 3:**
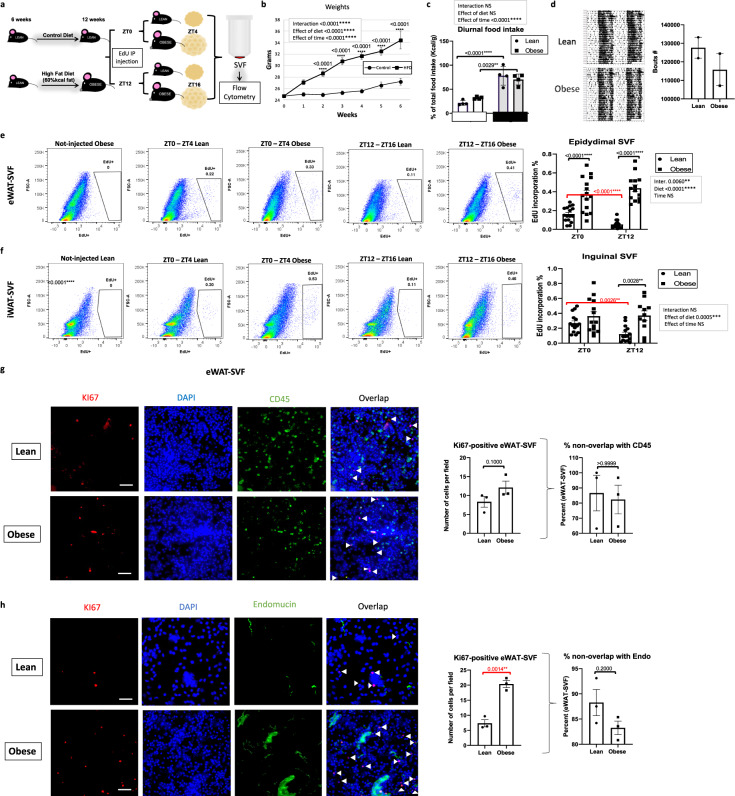
High-fat feeding disrupts the diurnal pattern of adipose stromal vascular cell proliferation in vivo. **a** Model of diet-induced obesity: 5-ethynyl-2ʹ-deoxyuridine (EdU) was intraperitoneally (IP) injected at zeitgeber time (ZT) 0 or ZT12 to lean mice fed control or high-fat diet (HFD) ad libitum. Epididymal fat (eWAT) and inguinal fat (iWAT)-derived stromal vascular cell fractions (SVF) were isolated from fat pads collected 4 h following EdU injection. **b** Weekly weight gain in chow and HFD-fed mice (*n* = 16 animals per diet). **c** Percent change in total food intake (kcal/g mouse) in light (white bar) vs. dark (gray bar) of mice fed vivarium chow or HFD ad libitum (*n* = 4 animals/diet and light or dark period). **d** Diurnal locomotion of group-housed mice as measured by infrared sensors (left panel) and corresponding quantification of bouts of activity (right panel). *n* = 2 cages with group-housed mice per each diet. **e**, **f** Representative fluorescence activated cell sorting of EdU-positive cells in eWAT (**e**) and iWAT (**f**) SVF (left panels) isolated at ZT4 and ZT16, 4 h following a single EdU injection, and respective quantification (right panels) (*n* = 16 animals/diet and ZT). **g**, **h** Representative staining of KI67 (vs. CD45)-positive cells (**g**) or KI67 (vs. endomucin)-positive cells (**h**) in eWAT-SVF (left panels) (scale bar = 50 µm) and respective quantification (right panels) (*n* = 3 animals/diet). t White arrows indicate the non-overlapping KI67. For all bar graphs, circles and squares represent lean and obese mice, respectively. Data are represented as mean ± SEM. ^*^p < 0.05, ^**^p < 0.01, ^***^p < 0.001, ^****^p < 0.0001. Significance (*p* < 0.05) determined by two-way ANOVA and Sidak’s post hoc test in (**b**) [F time(19, 2289)=237.1 / F diet(1, 2289) = 1337] or Tukey’s post hoc test in (**c**) [F time(1, 12) = 65.12 / F diet(1, 12) = 0.04475], (**e**) [F time(1, 56) = 0.4699 / F diet(1, 56) = 90.16], and (**f**) [F time(1, 51) = 2.271 / F diet(1, 51) = 13.86]; and two-tailed Mann–Whitney *U* test in (**g**, **h**). Red asterisks reveal significance determined by two-tailed unpaired *t*-test.

To determine whether the diurnal pattern of cell proliferation changes specifically during the development of DIO, 6-week-old mice were fed HFD for 6 weeks prior to EdU injection, which resulted in continuous weight gain throughout the feeding (Fig. 3a, b). Because diet has been reported to affect diurnal patterns of energy intake^24^, and to avoid metabolic changes in adipose tissue potentially occurring in mice undergoing CLAMS analysis, a separate cohort of mice were placed in metabolic chambers to determine if in spite of ad libitum feeding conditions, the rhythmicity of energy intake was similar to that of chow-fed animals (Fig. 3c). In addition, group-housed mice were maintained in circadian cages equipped with infrared sensors, to test for normal rhythms in home cage locomotion during the weeks preceding the experiment (Fig. 3d). After the 6th week of HFD feeding, EdU was injected at ZT0 or ZT12 in lean and DIO mice and WAT depots were removed 4 h following injections. SVFs were isolated and analyzed for EdU incorporation. HFD-fed mice showed a pronounced upregulation in the number of dividing SVFs in both eWAT and iWAT compared to control mice, which surprisingly, was devoid of diurnal pattern in both eWAT and iWAT (Fig. 3e, f). Pooling of the data from both ZTs revealed that HFD increased the number of proliferating SVFs in both iWAT and eWAT, but unlike lean mice, iWAT SVF in DIO mice did not differ from eWAT SVF in the number of proliferative cells (Fig. S3c). To verify whether the loss of diurnal SVF proliferation was not merely the result of a phase-shift in DIO mice, additional time points were analyzed (Fig. S3d, e). Neither eWAT nor iWAT showed rhythmicity in SVF proliferation under DIO, in contrast to lean animals (Fig. S3e). Subsequent staining of cells for CD45 and CD31 revealed that the vast majority of EdU-positive cells in the context of DIO were of stromal origin, not staining for either hematopoietic or endothelial markers (Fig. S3f). This is consistent with other studies^40,45^ that demonstrate the capacity of HFD feeding to induce proliferation of pre-adipocytes. However, comparing the absolute numbers of EdU-positive cells that were also positive for CD45 suggests an increase in the number of immune cells in the context of DIO in both fat pads compared to lean mice (compare Fig. S3f to Figs. 1g and S2b). Based on the diurnal proliferation of APCs in eWAT specifically under lean conditions, we tested whether the increased proliferation under DIO in eWAT involved a large number of endothelial or immune cells. Freshly purified SVF cells from eWAT harvested at ZT0 were plated onto slides and stained using Ki67, CD45, or endomucin antibodies to look for overlap of Ki67 with these markers (Fig. 3g, h). While the number of Ki67 cells increased in obese mice, as expected, the percent overlap of Ki67 with endomucin or CD45 remained unchanged, with the majority of Ki67+ cells (80–90%) being negative for endomucin or CD45.

### Rhythmic energy intake is necessary for diurnal proliferation in WAT SVFs

**Fig. 4:**
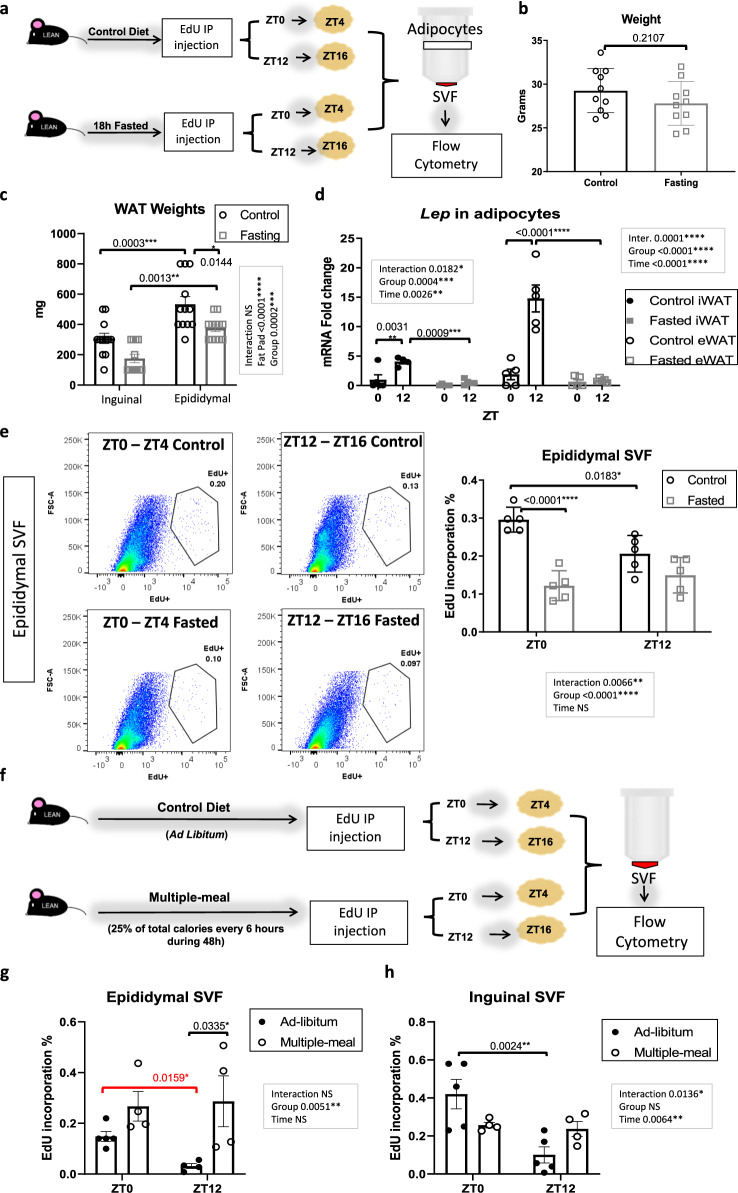
Ablation of rhythmic feeding destroys the diurnal pattern of adipose stromal vascular cell proliferation in vivo. **a** Experimental model: 5-ethynyl-2ʹ-deoxyuridine (EdU) was intraperitoneally (IP) injected at zeitgeber (ZT)0 or ZT12 to mice fed ad libitum chow or fasted for 18 h. Epididymal fat (eWAT) and inguinal fat (iWAT) stromal vascular cell fractions (SVF) and pure adipocytes were isolated from fat harvested 4 h following EdU injection (see also Fig. S3). **b** Weight of control and fasted animals (*n* = 10 animals/group). **c** Weight of fat pads in control vs. fasted mice (*n* = 12 animals/group). **d** qPCR reveals expression of leptin (*Lep*) in pure adipocytes from eWAT and iWAT in control vs. fasted groups (*n* = 6 animals/group). mRNA levels from the iWAT for fasted mice at ZT4 were set to 1. **e** Representative fluorescence activated cell sorting of EdU-positive cells from eWAT-SVF (left panels) isolated at ZT4 and ZT16, 4 h following EdU injection. Quantification, right panels (*n* = 5 animals/group). In (**b–d**) and (**e**), circles and squares represent fed ad libitum and fasted mice, respectively. **f** Experimental model for multiple-meal paradigm: EdU was administered at ZT0 or ZT12 to mice fed ad libitum or with total food intake divided into four aliquots, 25% being administered every 6 h throughout the diurnal cycle for 48 h. eWAT and iWAT-derived SVF were isolated from fat pads harvested 4 h following EdU injection. **g**, **h** Quantification of fluorescence activated cell sorting of EdU-positive SVF from eWAT (**g**) and iWAT (**h**) (*n* = 5 animals/group). **g**, **h** Black and white circles represent mice fed ad libitum and in multi-doses, respectively. Data are represented as mean ± SEM. ^*^p < 0.05, ^**^p < 0.01, ^***^p < 0.001, ^****^p < 0.0001. Significance (*p* < 0.05) determined by two-tailed unpaired *t*-test in (**b**) and for red asterisks; and two-way ANOVA followed by Tukey’s post hoc test in (**c**) [F fat pad(1, 44) = 36.08 / F group(1, 44) = 16.99], (**d**) [F group(1, 15) = 13.05 / F time(1, 15) = 20.35 (iWAT)] [F group(1, 31) = 37.19 / F time(3, 31) = 30.22 (eWAT)], (**e**) [F time(1, 16) = 2.735 / F group(1, 16) = 37.28], (**g**) [F time(1, 13) = 0.7485 / F group(1, 13) = 11.27], (**h**) [F time(1, 14) = 10.23 / F group(1, 14) = 0.06388].

Based on the loss of diurnal proliferation of SVFs in the context of DIO, we wondered if fasting would also alter the diurnal patterns of cell proliferation in vivo in lean animals. To study this, 3-month-old, lean mice fed normal chow and monitored for rhythmic activity were fasted for 18 h (Figs. 4a and S4a), which while not altering body weight, was sufficient to decrease basal glucose and fat pad mass (Figs. 4b, c and S4b). *Lep* expression in isolated adipocytes of both eWAT and iWAT was also reduced, though there was a more pronounced reduction in eWAT (Fig. 4d). In agreement with previous reports^46^, *Lep* expression was significantly higher in control eWAT and iWAT at ZT16 compared to ZT4, and fasting abolished the diurnal variation in *Lep* expression in eWAT (Fig. 4d). Following the fast, EdU was injected at either ZT0 or ZT12, after which pure adipocytes and SVFs were isolated from eWAT 4 h after EdU injection (Fig. 4a). When SVFs were analyzed for EdU incorporation, we found that fasting was sufficient to reverse the ZT0 peak in SVF proliferation in eWAT (Fig. 4e). Thus, both the timing and quality of nutrient input appear to be critical drivers of diurnal proliferation of APCs in WAT in vivo. To determine whether loss of rhythmic feeding with no caloric deprivation was sufficient to abolish diurnal SVF proliferation, daily energy intake of WT mice was determined by indirect calorimetry. Mice were administered 25% of their total daily energy intake every 6 h throughout the circadian cycle, to promote equal food consumption during the dark and light periods for 2 complete days, which was verified by an investigator (Fig. 4f). Loss of diurnal energy intake resulted in a complete loss of diurnal rhythms in SVF proliferation, resulting in similar numbers of proliferating cells at the end of the feeding and fasting phases in both adipose tissue depots (Fig. 4g, h). To verify that fasting did not merely shift rhythms of proliferation, the fasting experiment was repeated at the additional EdU injection time points of ZT6 and ZT18. EdU incorporation at these ZTs was not statistically significant between either ZTs or feeding conditions, revealing loss of rhythmicity in proliferation as opposed to a shift in rhythmicity (Fig. S4c).

**Fig. 5:**
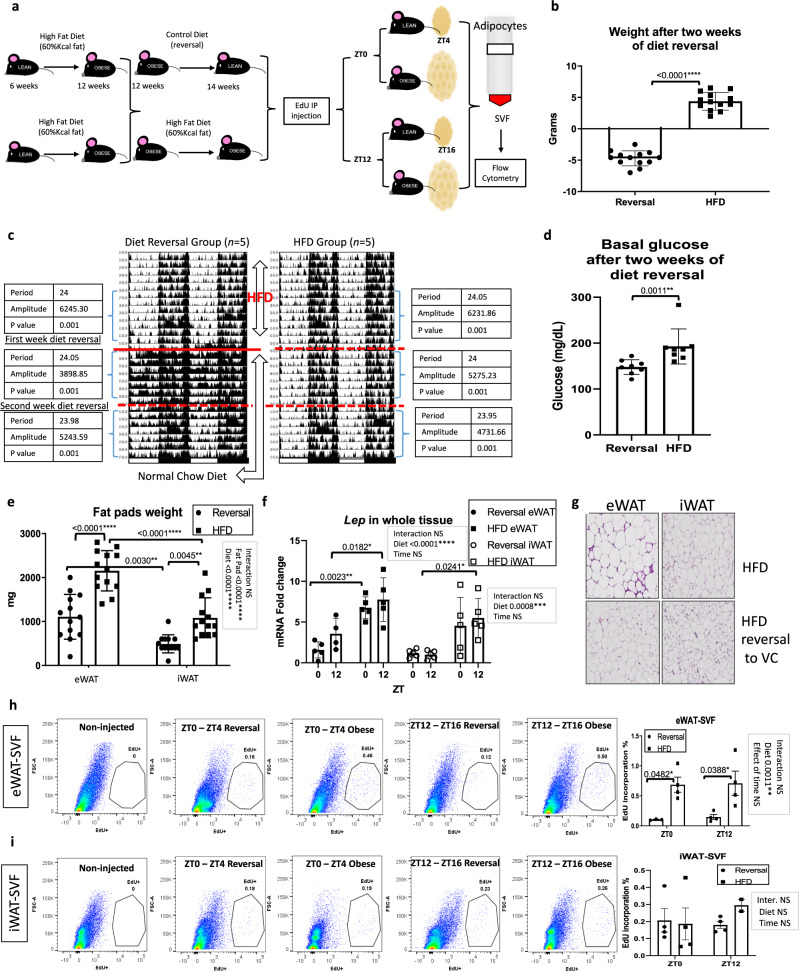
Diet reversal reduces proliferation without restoring diurnal patterns of WAT stromal vascular cell proliferation in vivo. **a** Experimental model: 5-ethynyl-2ʹ-deoxyuridine (EdU) was intraperitoneally (IP) injected at zeitgeber (ZT)0 or ZT12 to mice fed high-fat diet (HFD) followed by 2 weeks of chow feeding (reversal group) or continuous HFD. Whole epididymal WAT (eWAT) and inguinal WAT (iWAT), pure adipocytes, and stromal vascular cell fractions (SVF) were isolated. **b** Body weight of mice in each group (*n* = 13 animals/group). **c** Actograms of mice measured by infrared sensors. Straight and striped red lines indicate the start of diet reversal and elapsed weeks, respectively. Significance (*p* < 0.05) and parameters describing the fit determined by cosinor analysis using ClockLab software (Actimetrics). **d** Basal glucose of mice with or without diet reversal (*n* = 8 animals/group). **e** Weight of WAT pads with or without diet reversal (*n* = 13 animals/group). **f** qPCR reveals expression of *Lep* in both WAT depots with or without diet reversal at ZT4 and ZT16 (*n* = 5 animals/group). mRNA levels from the iWAT of diet-reversal mice at ZT4 were set to 1. **g** H&E staining of eWAT and iWAT with or without diet reversal (10X magnification, scale = 200 µm). Histology performed once from 3 biological replicates per condition. **h**, **i** Representative fluorescence activated cell sorting of EdU-positive SVF from eWAT (**h**) and iWAT (**i**) isolated at ZT4 and ZT16, 4 h following a single EdU injection (left panels). Quantification, right panels (*n* = 4 animals/group). Data are represented as mean ± SEM. ^*^p < 0.05, ^**^p < 0.01, ^***^p < 0.001, ^****^p < 0.0001. Significance (*p* < 0.05) determined by two-tailed unpaired *t*-test in (**b**); two-tailed Mann–Whitney *U* test in (**d**); two-way ANOVA followed by Tukey’s post hoc test in (**e**) [F fat pad(1, 48) = 51.43 / F diet(1, 48) = 48.65], (**f**) [F time(1, 15) = 3.080 / F diet(1, 15) = 30.68 (eWAT)] [F time(1, 16) = 0.1675 / F diet(1, 16) = 17.11 (iWAT)], (**h**) [F time(1, 11) = 0.06920 / F diet(1, 11) = 19.12], (**i**) [F time(1, 10) = 0.3203 / F diet(1, 10) = 0.4203]. For all bar graphs, black circles and squares represent mice fed chow diet reversal or HFD, respectively (white circles and squares for iWAT in (**f**)).

To determine whether HFD reversal can restore levels and rhythmicity of SVF proliferation in vivo, mice fed with HFD for 6 weeks were either maintained on the diet or, alternatively, placed back on normal chow diet for 2 weeks (Fig. 5a). This feeding paradigm was chosen to maintain consistency in the time of HFD exposure for mice in Fig. 3a. After 2 weeks of diet reversal, mice on diet reversal lost body weight and remained rhythmic, as assessed by infrared sensor detection of home cage activity (Fig. 5b, c). Loss of body weight was accompanied by a reduction of blood glucose and fat mass (Fig. 5d, e) and decreased leptin expression in both depots, though rhythmicity in *Lep* expression was not restored by diet reversal (Fig. 5f). Two weeks following diet reversal, HFD and diet-reversal mice were injected with EdU at ZT0 or ZT12 and WAT pads were removed for subsequent analysis. Hematoxylin and eosin (H&E) staining revealed reduced adipocyte size in both eWAT and iWAT of mice on diet reversal (Fig. 5g), but an overall reduction in proliferating SVFs was observed only in eWAT as measured by EdU-positive cells (Fig. 5h). Although eWAT SVF proliferation was completely reversed to control diet levels observed in previous experiments, diet reversal was not sufficient to restore the diurnal patterns of cell proliferation (Fig. 5h). Importantly, HFD reversal did not reduce the consistently higher basal proliferation in SVF of iWAT (Fig. 5i), possibly due to the increased number of proliferative endothelial cells that oscillate in SVF compared to eWAT. Purified adipocytes prepared from control and diet-reversal mice revealed reduced *Lep* expression in the adipocyte fraction of eWAT in diet-reversal mice, though the diurnal variance in *Lep* expression in purified adipocytes was not restored to control levels (Fig. S5a, compare to Fig. 4d), similar to the *Lep* expression results in the whole tissue (Fig. 5f). Consistent with a downregulation of adiponectin (*Adipoq*) in obesity^47,48^, diet reversal increased *Adipoq* in both eWAT and iWAT, particularly at the ZT16 time point (Fig. S5b). Lipoprotein lipase (*Lpl*) expression was also altered in both WAT depots after 2 weeks of diet reversal (Fig. S5c). To provide further evidence that diet reversal alters proliferation in eWAT SVF, mRNA from eWAT SVF from both groups was analyzed for *Ki67* expression. *Ki67* expression was greatly reduced in eWAT SVF of animals having undergone diet reversal, consistent with the loss of EdU incorporation in vivo (Fig. S1d). Pooled data of EdU incorporation from both ZTs reinforces the fact that depot-specific differences exist in proliferative capacity of SVF cells (Fig. S5e). In summary, diurnal SVF proliferation is highly diet responsive, and HFD may result in sustained diurnal disruption of SVF proliferation in eWAT SVFs.

### The circadian clock controls proliferation of SVFs

**Fig. 6:**
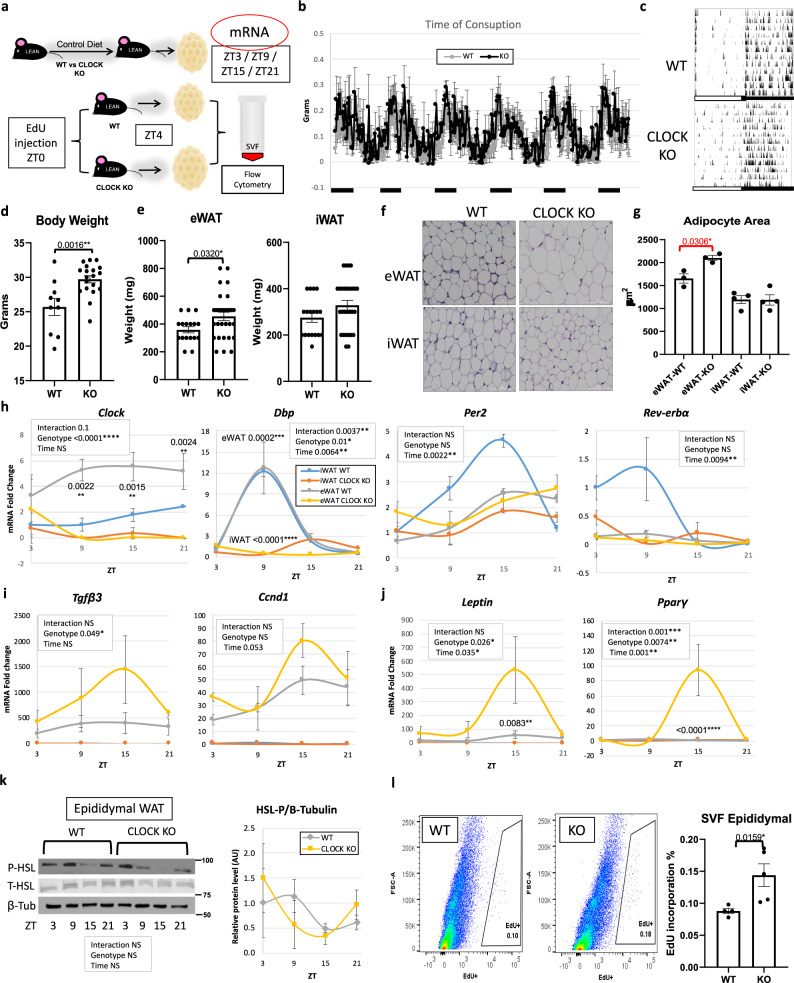
*Clock*-deficiency induces proliferative markers in epididymal fat (eWAT). **a** Models of *Clock*-deficiency: eWAT and inguinal fat (iWAT) was collected every 6 h or 5-ethynyl-2ʹ-deoxyuridine (EdU) was administered at zeitgeber time (ZT) 0 and eWAT and iWAT stromal vascular cell fractions (SVF) were isolated from fat 4 h later. **b** Energy intake of *Clock* KO and WT littermates under 12 h light/12 h dark and ad libitum feeding (*n* = 6 animals/genotype). **c** Home cage locomotion of *Clock* KO and WT littermates measured by infrared sensors. Bottom white and black lines indicate the light or dark periods, respectively (**b**, **c**). **d** Weights of *Clock* KO (*n* = 19 animals) and WT littermates (*n* = 10 animals). **e** Fat pad weights of *Clock* KO (*n* = 29 animals) and WT littermates (*n* = 18 animals). **f** Representative H&E staining of eWAT and iWAT from *Clock* KO and WT littermates (20X magnification, scale = 100 µm) and quantification (*n* = 3 animals/genotype, eWAT and *n* = 4 animals/genotype, iWAT examined over two independent experiments) (**g**). **h**–**j** RT-PCR reveals expression of clock (**h**), proliferation (**i**), and adipogenesis (**j**) markers in iWAT (blue = WT, orange = KO) (see also Fig. S7) and eWAT (gray = WT, yellow = KO), from *Clock* KO and WT littermate mice over 24 h (*n* = 3 animals/ ZT). mRNA levels in iWAT for WT at ZT3 set to 1 for all genes. **k** Western blot reveals expression of phosphorylated hormone-sensitive lipase (P-HSL), total HSL (T-HSL) and β-Tubulin throughout the circadian cycle (*n* = 3 animals/ZT) in eWAT (left panel). Quantification, right panel (gray = WT; yellow = KO). Protein levels from WT mice at ZT3 set to 1. **l** Representative in vivo fluorescence activated cell sorting of EdU-positive cells from eWAT (left panel) SVF isolated at ZT4, 4 h following a single EdU injection. Right panel: respective quantification (*n* = 6 animals for Clock KO and *n* = 4 for WT littermate animals). Data are represented as mean ± SEM. ^*^p < 0.05, ^**^p < 0.01, ^***^p < 0.001, ^****^p < 0.0001. Significance (*p* < 0.05) determined by two-tailed, unpaired *t*-test in (**d**, **e**) and for red asterisks; two-tailed Mann–Whitney *U* test in (**g**, **l**); and two-way ANOVA followed by Sidak’s post hoc test in (**h–k**).

The importance of the circadian transcriptional activator, BMAL1, in adipose tissue function has been demonstrated via the inducible knockout of *Bmal1* specifically in adipocytes^8^. Loss of adipocyte-specific BMAL1 in mouse WAT results in arrhythmic release of polyunsaturated fatty acids and eventually, adiposity via the loss of rhythmicity in daily energy intake. Though our data in WT mice reveal that rhythmic feeding is necessary for diurnal proliferation of SVFs in eWAT, we tested whether the circadian clock in addition to rhythmic energy intake contributes to diurnal changes in SVF proliferation. To test this, we used global *Clock*-deficient mice^49^, which maintain central rhythmicity, but have peripheral arrhythmicity due to loss of CLOCK in all cell types^50,51^. To determine whether CLOCK deletion altered diurnal properties of adipose tissue, eWAT and iWAT of *Clock*-deficient mice (*Clock* KO mice) along with their littermate controls were harvested every 6 h throughout the circadian cycle (Fig. 6a). *Clock* KO mice are considered to be rhythmic in the central nervous system, though have deficiencies in peripheral rhythmicity^49–51^, likely due to the limited expression of the BMAL1 partner, NPAS2 in the periphery compared to the brain. To confirm rhythmicity in overall circadian activity, a subset of *Clock* KO and WT mice were subjected to indirect calorimetry and circadian monitoring by infrared sensors. Energy intake patterns were similar between *Clock* KO and WT littermates, and both genotypes maintained rhythmicity in entrained 12 h light/12 h dark conditions (Fig. 6b, c). As previously reported^52^, *Clock*-deficient mice gain weight throughout aging, even under chow-feeding conditions, which we observe is in part due to an increase in eWAT mass as early as 3 months of age (Fig. 6d, e). H&E staining of adipose tissue revealed that loss of CLOCK resulted in larger adipocytes in eWAT (Fig. 6f, g). To determine whether loss of CLOCK alters gene expression related to cell proliferation in adipose tissue, whole-WAT depots from *Clock*-deficient mice were analyzed for gene expression throughout the 24 h cycle. In addition to the absence of *Clock* in KO mice, *Dbp* was greatly reduced and devoid of oscillatory expression in *Clock*-deficient mice, and *Per2* and *Rev-erba*, were also greatly reduced in the eWAT of *Clock* KO mice (Figs. 6h and S6a). On the other hand, markers of cell proliferation and adipogenesis were generally increased in eWAT in the absence of CLOCK (Fig. 6i, j), though changes in cell proliferation and adipogenesis genes were not present in whole-iWAT tissue (Fig. S6b, c). Congruent with these data, when pure adipocytes from *Clock* KO and WT mice were isolated and counted from eWAT and iWAT, the total number of adipocytes in *Clock* KO eWAT trended higher compared to controls, while iWAT adipocyte number appeared invariant (Fig. S6d). To determine whether differences in eWAT morphology in *Clock*-deficient mice might be attributed to changes in lipolysis, we assessed phosphorylation of hormone-sensitive lipase (HSL), which is a general indicator of its activity in adipose fatty acid release^53^. Though *Hsl* has been reported to contain E box elements responsive to the CLOCK:BMAL1 heterodimer^54^, analysis of HSL phosphorylation revealed a similar diurnal pattern in *Clock*-deficient and WT mice, suggesting that lipolysis is not substantially altered in *Clock*-deficient mice in vivo (Fig. 6k). Finally, to determine whether *Clock*-deficient SVF also have a greater proliferative capacity in vivo, WT and *Clock*-deficient mice were injected with EdU at ZT0, the time of peak incorporation in WT animals. Isolation of SVFs from eWAT harvested 4 h following EdU injection revealed more proliferating cells in *Clock*-deficient mice, revealing that presence of the circadian clock protein CLOCK controls normal proliferation and adipogenesis in eWAT (Fig. 6l).

**Fig. 7:**
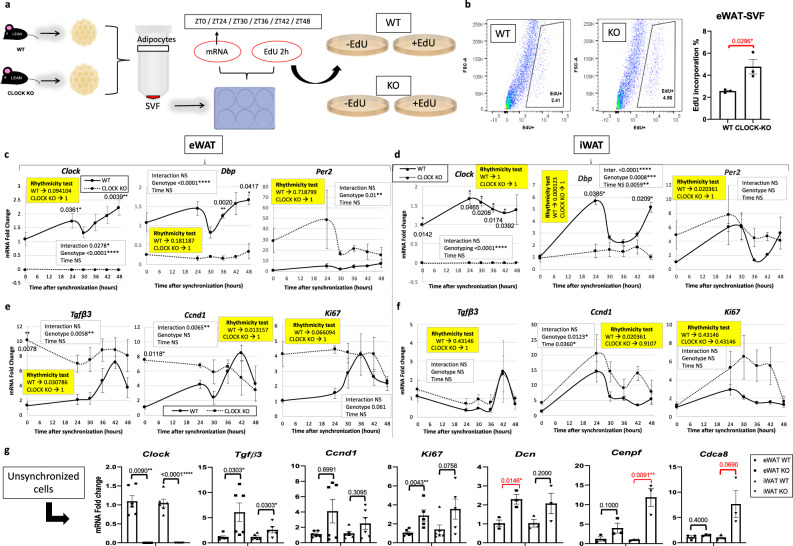
*Clock*-deficiency results in higher levels of epidydimal fat (eWAT) stromal vascular cell fraction (SVF) proliferation in vitro. **a** In vitro models of *Clock* deficiency: eWAT and inguinal fat (iWAT) SVF and pure adipocytes were isolated from *Clock* KO and WT littermate mice for further analysis (*n* = 3 technical replicates, from each of 2 biological replicates), and serum shocked eWAT-SVF isolated from *Clock* KO or WT littermates (*n* = 2 technical replicates, from each of 3 biological replicates) were incubated with EdU for 2 h. **b** Representative in vitro fluorescence activated cell sorting of EdU-positive cells from eWAT-SVF (left panels) and quantification (right panel) (*n* = 3 animals/genotype). **c–f** qPCR reveals mRNA abundance of circadian clock (**c**, **d**) and proliferation (**e**, **f**) genes in serum shocked eWAT- (**c**, **e**) and iWAT-derived (**d**, **f**) SVF from *Clock* KO (dashed lines) vs. WT littermates (solid lines) (representative experiment, *n* = 3 technical replicates). mRNA levels for WT mice at ZT0 were set to 1. Yellow boxes indicate the significance (*p* < 0.05) for rhythmicity test. **g** RT-PCR reveals mRNA abundance of *Clock* and proliferation genes in cultured, unsynchronized epididymal and inguinal SVF from *Clock* KO vs. WT littermates (*n* = 3 technical replicates, from each of 2 biological replicates). mRNA levels for WT mice at ZT0 were set to 1. Circles (WT) and squares (KO) represent eWAT, upward-facing triangles (WT) and downward-facing triangles (KO) represent iWAT. Data are represented as mean ± SEM. ^*^p < 0.05, ^**^p < 0.01, ^***^p < 0.001, ^****^p < 0.0001. Significance (*p* < 0.05) determined by two-tailed Mann–Whitney *U* test in (**g**); two-tailed unpaired *t*-test for red asterisks and two-way ANOVA and Sidak’s post hoc test in (**c**–**f**). For all bar graphs, circles and squares represent WT or CLOCK KO mice, respectively (triangles facing up or down, respectively, for iWAT in (**g**)).

To determine whether CLOCK deficiency affected markers of proliferation and adipogenesis specifically in SVFs of WAT, eWAT and iWAT was removed from WT and *Clock*-deficient littermate mice at ZT4 and fractionated into the SVF and adipocyte fractions for gene expression analysis or plated to monitor proliferation in vitro (Fig. 7a). SVFs from *Clock* KO and WT mice exposed to EdU for 2 h at various time points following a serum shock revealed a large number of EdU^+^ cells in *Clock*-deficient eWAT SVFs compared to WT eWAT SVFs, though proliferation in iWAT SVF trended higher but was not significant (Figs. 7b and S7a). Loss of CLOCK in the eWAT resulted in robust *Lep* expression at ZT4 in MA (Fig. S7b). Serum shock of cultured SVFs from *Clock* KO mice revealed significant changes in both circadian clock and pro-mitogenic genes (Fig. 7c–f). Specifically, serum shock induced rhythmicity in *Tgfβ3, Ccnd1*, and *Ki67* in SVF isolated from eWAT of WT mice, while *Clock*-deficient SVFs showed no rhythmicity in the expression of any of these genes in either iWAT or eWAT (Fig. 7e, f). Similar to what was observed in vivo, unsynchronized SVF from eWAT showed pronounced changes in mitogenic gene expression, though for some genes, changes in expression did not reach significance (Fig. 7g). In summary, CLOCK contributes to proliferation in WAT SVF, though depot-specific differences were noted in vivo, suggest additional circulating factors may be involved in circadian-regulated adipose tissue growth and expansion.

**Fig. 8:**
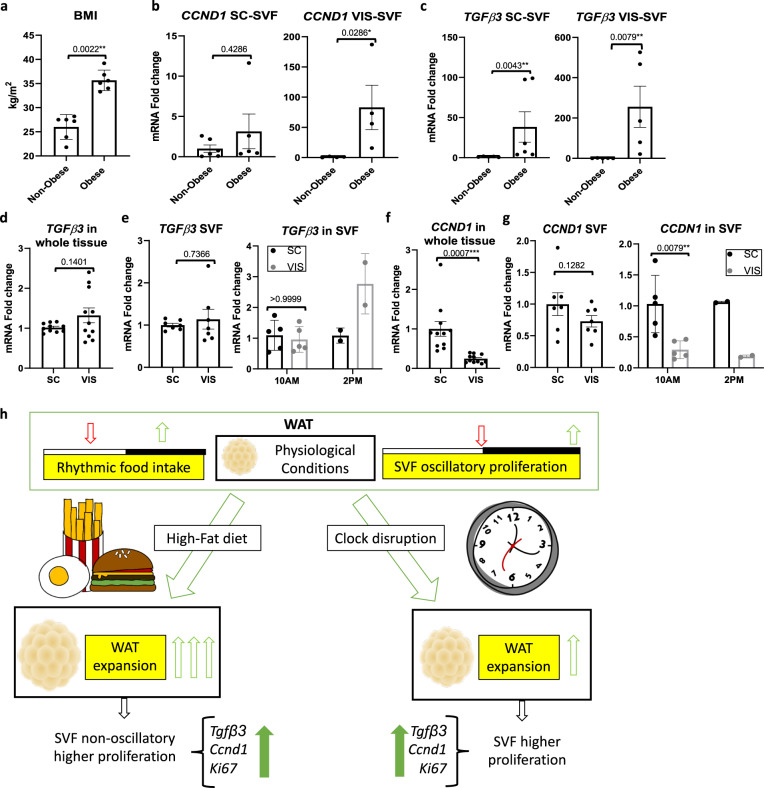
*TGFβ3* and *CCND1* expression varies across human adipose tissue depots. **a** Body mass index (BMI) from non-obese and obese human patients undergoing prostatectomy intervention (*n*  =  6 patients/group). **b**, **c** RT-PCR analysis reveals expression of *CCND1* (**b**) and *TGFΒ3* (**c**) in periprostatic visceral (VIS) or abdominal subcutaneous (SC) SVF prepared from prostatectomy intervention subjects (*n* = 6 patients/group). mRNA levels from SC fat in lean individuals set to 1. **d** RT-PCR analysis reveals expression of *TGFΒ3* in omental (VIS) and subcutaneous (SC) tissue from obese patients undergoing bariatric surgery (*n* = 12 patients/fat pad). mRNA levels from the SC fat set to 1. **e** RT-PCR analysis reveals expression of *TGFΒ3* in VIS- and SC-derived SVF (*n* = 8 patients/fat pad) (left panel) and isolated SVF in the morning (approximately 10:00) (*n* = 6 patients/fat pad) vs. afternoon (approximately 14:00) (*n* = 2 patients/fat pad) (right panel) from patients undergoing bariatric surgery. mRNA levels from the SC (left panel) or the SC at 10:00 (right panel) were set to 1. **f** RT-PCR analysis reveals expression of *CCND1* in VIS and SC fat tissue from patients undergoing bariatric surgery (*n* = 12 patients/fat pad). mRNA levels from the SC fat were set to 1. **g** RT-PCR analysis reveals expression of *CCND1* in VIS and SC isolated SVF (*n* = 8 patients/fat pad) (left panel) taken at 10:00 (*n* = 6 patients/fat pad) or 14:00 (*n* = 2 patients/fat pad) (right panel) from obese patients subjected to bariatric surgery. mRNA levels of all SC (left panel) or the SC at 10:00 (right panel) were set to 1. **h** Working model of diurnal progenitor proliferation. Data are represented as mean ± SEM. ^*^p < 0.05, ^**^p < 0.01, ^***^p < 0.001, ^****^p < 0.0001. Significance (*p* < 0.05) determined by two-tailed Mann–Whitney *U* test in (**a–c**, **e**, **g**); two-tailed unpaired *t*-test in (**d**, **f**).

Based on the increased expression of pro-proliferative *Tgfβ3* in the context of HFD feeding, we wondered to what extent human visceral adipose tissue might function similarly to the murine fat in diurnal proliferative capacity. We analyzed non-cultured SVFs from different depots of human fat from non-obese but overweight individuals (average body mass index (BMI) = 26.02 ± 1.05) and obese patients (average BMI = 35.67 ± 0.87) (Fig. 8a). Consistent with the rodent data, *TGFβ3* and *CCND1* expression was considerably elevated in SVFs of periprostatic (visceral, VIS-SVF) and subcutaneous (SC-SVF) WAT in obese compared to overweight but non-obese individuals (Fig. 8b, c), suggesting that human WAT may have similar proliferative potential during the circadian cycle that differs in obese vs. non-obese conditions. Because serum shock of SVFs resulted in rhythmic *Tgfβ3* expression in cultured murine WT cells and higher expression in eWAT compared to iWAT in mice (Fig. S8a), omental visceral (VIS) and subcutaneous (SC) adipose tissue were obtained from obese individuals undergoing gastric sleeve surgery in the morning or afternoon (Table S1), and SVFs were isolated and analyzed for *TGFβ3* expression. Similar to the observations of *TGFβ3* expression in the periprostatic fat of obese patients compared to *TGFβ3* expression in subcutaneous fat of the same individuals (Fig. 8c), *TGFβ3* expression in fat from gastric sleeve patients trended higher in whole tissue and SVFs from VIS WAT compared to SC WAT (Fig. 8d, e), particularly in patients undergoing surgery in the afternoon compared to the morning (Fig. 8e, right panel), though fasting conditions prior to surgery could have some effect on the diurnal differences in human patients. Thus, consistent with *Tgfβ3* expression in rodent tissue (Fig. S8a), *TGFβ3* expression is greatly enhanced under conditions of obesity in visceral fat, suggesting that human *TGFβ3* may display a similar time-of-day role in the proliferation of adipose tissue under lean conditions. Though *TGFβ3* has been demonstrated to be pro-proliferative in the context of pre-adipocytes^3^, *Ccnd1* is a marker of active cell proliferation. Similar to our rodent data, where *Ccnd1* is generally higher in iWAT compared to eWAT (Fig. S8b) we found that *CCND1* was also elevated in human SC WAT compared to VIS WAT of patients undergoing gastric bypass surgery (Fig. 8f). This depot-specific change was reflected by further fractionation and analysis of SVFs from the corresponding depots and was consistent across morning and afternoon harvest times (Fig. 8g, right panel). Thus, human SVF shows depot-specific patterns of pro-proliferative genes that appear, like our preclinical data, to be elevated in the context of obesity.

## Discussion

Prior studies addressing circadian rhythmicity in human adipose tissue have revealed the presence of robust rhythmicity in whole WAT, and also in subcutaneous adipose-derived stem cells^4,55,56^. Specifically, prior studies using isolated human SVF from inguinal fat have demonstrated both the inducibility and robustness of the circadian clock in these cells^55^. The data presented here reveal a previously unappreciated diurnal aspect of adipose tissue rhythms. Specifically, these results reveal the remarkable capacity for a subset of APCs to undergo diurnal proliferation in vivo. While the circadian clock contributes to the diurnal aspect of these proliferative signals particularly in eWAT, as demonstrated by the *Clock*-deficient mice, rhythmicity in energy intake is also a critical circadian component of this oscillation in vivo (Fig. 8h). Cell transplantation studies have revealed that visceral WAT adipogenesis is regulated by the microenvironment as opposed to cell-intrinsic mechanisms^57^. Thus, we hypothesize that circulating circadian factors, whether in the serum or contributed via rhythmic sympathetic innervation of visceral WAT may be responsible for driving diurnal rhythms in pre-adipocyte proliferation. These factors could include but are not limited to rhythmic circulating neuroendocrine hormones, such as growth hormone^58^ and glucocorticoids^59^, both of which are known to target adipose tissue, affecting adipose tissue function and mass^60,61^.

HFD is known to abolish most metabolite rhythms in the serum^6^, which could be one possible explanation for the loss of rhythmicity in APC proliferative capacity under HFD conditions. Our data reveal that while rhythmic energy intake is important for diurnal rhythms of cell proliferation within the SVF compartment under conditions of energy balance, HFD both elevates proliferation within WAT, and destroys its diurnal patterns, even under conditions of rhythmic energy intake. The total loss of rhythmicity under HFD was somewhat surprising, considering that several proliferation markers were induced in a circadian fashion under conditions of HFD. There are several possible factors causing this total loss; however, staining and sorting of cells for CD45 and CD31 suggest that the loss of diurnal proliferation is directly related to the elevated proliferation of cells of stromal origin that no longer show a diurnal pattern. This is an important observation in lieu of previous studies demonstrating the circadian regulation of hematopoietic progenitors in the circulation^62,63^, a variable that should be considered in the context of diurnal proliferation within the SVF of adipose tissue. Interestingly, under conditions of HFD, CD45^+^ cells maintained a low-amplitude diurnal proliferation, suggesting a circadian defect specifically in the adipocyte progenitors themselves. This would be consistent with diet-reversal experiments, in which rhythmicity in SVF proliferation was not regained 2 weeks after diet reversal, nor was rhythmicity in *Lep* expression in adipocytes, though body and adipose tissue weights were substantially decreased (Fig. 5). Studies have shown that in formerly obese mice, adipose tissue macrophages can proliferate and remain in adipose tissue for up to 6 months after weight loss, and as a result, insulin sensitivity of WAT remains compromised^42^. The fact that the majority of Ki67-positive cells in DIO eWAT did not overlap with CD45^+^ suggests that macrophages were not the primary cell type induced to proliferate in DIO, nor a major cause of the decrease in proliferation under diet reversal. An interesting outcome of the DIO model, however, was the observation that the circadian clock and cell proliferation became uncoupled, evident by several proliferation markers that were not only induced but also became either de novo rhythmic or gained higher amplitude rhythms. This diet-induced gain of rhythmicity in gene expression is reminiscent of what has been observed in other tissues in response to HFD. For example, PPARG and its targets acquire robust rhythms in the liver, likely an adaptive response to assist in storing lipids under conditions of excess circulating fatty acids^6,21^. Interestingly, there is some evidence that the circadian clock may restrain cell cycle progression by direct coupling^64,65^, a process that is thought to be disrupted in cancer^66^, but may be in place in APCs to control diurnal patterns of proliferation under conditions of energy balance. HFD has also been shown to modulate chromatin and expression of clock genes via activation of the pro-inflammatory NF-kB pathway, a possible mechanism for circadian perturbation in this context^64^. Interestingly, though HFD exposure can initially induce diurnal changes in energy intake^5,24,67^, at later time points rhythmicity in energy intake is largely regained^21,67^ (also see Fig. 3c). Thus, HFD may impair diurnal regulation of proliferation in APCs early on, and in a manner which is not restored by a return of rhythmic energy intake. In the context of diet reversal after HFD, it would be interesting to determine whether longer periods of diet reversal would be sufficient to restore diurnal patterns of SVF proliferation, or whether this diurnal regulation remains permanently disrupted.

While feeding is clearly an important driver of diurnal WAT SVF proliferation, a fully functional CLOCK:BMAL1 transcriptional complex appears to be necessary for maintaining normal proliferation of SVFs in vitro and in vivo. Though some circadian mutant mice show aberrations in diurnal lipolysis of adipose tissue^54^, *Clock*-deficient mice had similar diurnal patterns of HSL phosphorylation in the eWAT compared to WT littermate mice. Thus, based on the heightened proliferative capacity of *Clock*-deficient eWAT SVFs in vitro and in vivo, the increased fat mass, and increased MA number, we conclude that larger visceral fat in the *Clock*-deficient mice is a combination of increased pre-adipocyte proliferation and adipogenesis as well as hypertrophy (Figs. 6f and S6d). Though the depot-specific effects of CLOCK are not fully understood, the fact that eWAT was more affected by loss of CLOCK than iWAT in vivo, is consistent with one study that suggests a correlation between the expression of clock genes (*Bmal1*, *Per2*, and *Cry1*) in human peripheral blood mononuclear cells and visceral fat mass, while there was no correlation between any the expression of any clock gene and subcutaneous fat mass^68^. Though proliferation was enhanced in cultured eWAT SVFs of *Clock*-deficient mice, whether *Clock*-deficient pre-adipocytes would begin to proliferate rhythmically in a WT host with normal rhythmicity, considering the environmental contribution to pre-adipocyte proliferation in not known^42^. Based on the altered circadian metabolome within the serum of *Clock*-deficient mice^52^, it is possible that rhythmic cues controlling adipocyte proliferation may be altered in these animals. Similarly, various neuroendocrine hormones have been shown to be regulated by the circadian clock, which could also affect proliferation of adipose stromal cells^69^. Depletion of *Clock* specifically in APCs is needed to fully understand the cell autonomous vs. non-autonomous effects of CLOCK in the context of progenitor proliferation.

An interesting observation from these data is that under lean conditions, pre-adipocyte proliferation within iWAT is slightly higher basally than in eWAT. Surprisingly, while eWAT proliferation was dramatically reduced under conditions of diet reversal, iWAT expression of *Ki67* in SVFs and cell proliferation in vivo was unaffected by HFD reversal. Further studies are needed to determine whether this may be due to a persistent contribution of APC proliferation, or alternatively endothelial cell proliferation in the subcutaneous fat, which is known to be more heavily vascularized than visceral adipose tissue. Regardless, other studies support a direct role for APC proliferation in subcutaneous adipose tissue mass. For example, pro-apoptotic peptides that deplete APCs suppress adipose tissue expansion in mice fed HFD^70,71^. Recent single-cell RNA-seq data have shown that these peptides function by depletion of proliferating APCs, which also happen to be DPP4-negative under conditions of energy balance, and thus are unlikely to become fibroblasts as opposed to new adipocytes^72^. Thus, though adipocyte hypertrophy is a key mechanism of adipose tissue expansion, we propose that over time, diurnal regulation of APC proliferation may be a key mechanism by which healthy adipose tissue mass is regulated under conditions of energy balance.

In summary, these data provide a diurnal basis for sustained adipose tissue SVF proliferation, driven largely by APCs in eWAT that is driven by feeding and the circadian clock. Loss of rhythmic feeding, nutrient challenge, or loss of CLOCK expression are all sufficient to abolish this diurnal pattern, suggesting that like some other peripheral cell types, the circadian clock of pre-adipocytes is highly vulnerable to disruption by environmental zeitgebers. These data provide insights regarding the potential role of the circadian clock in adipose tissue mass regulation, and suggest that rhythmic control of pre-adipocyte proliferation may be an essential regulator of not only adipocyte mass but also metabolic health in the long term.

## Methods

### Animals

Procedures related to the care and use of mice were reviewed and approved by the McGovern Medical School, UT Health Science Center Animal Care and Use Committee (protocols AWC-18-0077, CCE-130701, and AWC-18-0066) and by the local authorities of the state of Saxony, Germany, as recommended by the responsible local animal ethics review board (Landesdirektion Leipzig, TVV15/16). Animal experiments were carried out in adherence to guidelines. Male C57BL/6J mice (Jackson Laboratories, #000664) and *Clock*-deficient (*Clock*^*−/−*^) and WT littermate animals (provided by Dr. David Weaver) (Worcester, MA)^49^ between 3 and 4 months of age (unless otherwise indicated) were used across different experimental designs. C57Bl/6N mice (Charles River) maintained in the University of Leipzig vivarium and used where indicated in the “Experimental design” section. Heterozygous mating pairs were used for breeding so that WT and *Clock*^*−/−*^ littermates could be generated for experiments. Unless otherwise indicated, mice were allowed to drink and eat ad libitum, one of the two diets: vivarium chow (VC) (13% kcal from fat, LabDiet #5R53) or high-fat diet (HFD) (60% kcal from fat, Research Diets #D12492) to induce obesity. Animals were maintained on a 12 h light (L)/12 h dark (D) schedule unless otherwise indicated. For all experiments, zeitgeber (ZT) 0 refers to light onset in the room, corresponding to the beginning of the animals’ sleep/fasting phase. ZT12 refers to the onset of darkness, the beginning of the animals’ active/feeding phase. In general, at the time of harvest, inguinal adipose tissue (iWAT) and epididymal adipose tissue (eWAT) were taken from lean and obese WT animals (C57Bl6/J) or *Clock*-deficient animals throughout the circadian cycle. Excised tissues were either frozen at −80 °C for future processing of the whole tissue or alternatively, processed immediately for the isolation of stroma vascular fraction cells (SVF), and/or mature adipocytes (MA).

#### Experimental design 1

Figs. 1, 2, S1–S3, and S8: Male C57BL/6J mice (JAX 000664) were exposed to VC or HFD starting at 8 weeks of age. Animals were euthanized after 18 weeks on the diet at ZT0, ZT4, ZT8, ZT12, ZT16, ZT20, and ZT24 (*n* = 4 per diet/ZT). A small chunk of iWAT and the eWAT were removed and immediately placed in liquid nitrogen for further analysis (Figs.1a, b and S8a, b; and S1A-B), while remaining adipose tissue was used to isolate the SVF (S3B).

#### Experimental design 2

Figs. 1, 2, S1, and S2: Male C57BL/6J mice (JAX 000664) were injected at ZT0 or ZT12 (*n* = 17 per ZT), or at ZT6 or ZT18 (*n* = 12 per ZT) with 5ethynyl-2ʹ-deoxyuridine (EdU) (Cayman #20518) at a concentration of 100 mg/kg of body weight. Mice were euthanized at ZT4 or ZT16 or at ZT10 or ZT22, 4 h following the single EdU injection. Mice non-injected were used for each time point as a negative control (*n* = 4). Following euthanasia, iWAT and eWAT were quickly removed and fractionated into SVF and MA as described in the “White adipose tissue fractionation” section. The SVF pellet was washed, fixed and directly labeled with Click-iT EdU Alexa Fluor 647 kit (Invitrogen C10424) following manufacturer directions, and subsequently analyzed by cell cytometry as described in the “Flow cytometry” section. A separate cohort of age-matched mice fed the same diet ad libitum were analyzed by infrared sensors (Fig. 1c) to validate diurnal and circadian activity as described in the “Circadian activity” section, and placed into metabolic cages after 5 weeks of feeding to measure diurnal food intake patterns (Fig. 1d) as described in the “Metabolic phenotyping” section. A separate cohort was used to factor out potential weight loss that commonly occurs in metabolic cages.

#### Experimental design 3

Figs. 1 and S2: Male C57BL/6N background mice were injected at ZT0 or ZT12 (*n* = 4 per ZT) with EdU as described above and then euthanized at ZT4 or ZT16, respectively. Following euthanasia, the SVF from iWAT and eWAT were isolated as described in the “White adipose tissue fractionation” section. The SVF pellet was washed and fractionated into adipose progenitor cells (APC^+^) or non-adipose progenitor cells (APC^−^) with the adipose tissue progenitor isolation kit (Miltenyi Biotec 5200501358) following manufacturer directions. Cell fractions then were fixed and labeled with Click-iT EdU Alexa Fluor 647 kit (Invitrogen C10424) and subsequently analyzed by cell cytometry. A separate cohort of age-matched mice were sacrificed at ZT0 or ZT12 and both APC^+^ and APC^−^ fractions were isolated from the iWAT and eWAT and immediately frozen at −80 °C for further gene expression analysis.

#### Experimental design 4

Figs. 2 and S2: Male C57BL/6J mice were exposed to VC or HFD starting at 6 weeks of age. After 6 weeks on diet, animals were injected at ZT0 or ZT12 with EdU or vehicle as described above and then euthanized at ZT4 or ZT16, respectively (*n* = 12 per diet/ZT). iWAT and the eWAT were removed and immediately fractionated and processed as in the experimental design 2. Animals were analyzed in cages equipped with infrared sensors to study diurnal and circadian activity during the 6 weeks feeding paradigm (Fig. 3d). A separate cohort of age-matched mice subjected to the same feeding regimens were sacrificed at ZT0 and the SVF from eWAT was isolated and subjected to cytocentrifugation for further staining analysis as described in the “Fluorescent staining” section.

#### Experimental design 5

Fig. S3d–f: Male C57BL/6 J background mice were exposed to VC or HFD starting at 6 weeks of age for 17 weeks, to mimic the body weights of mice used in experimental design 1. Mice (*n* = 4/ZT) were given a single injection of EdU or vehicle at ZT0, ZT6, ZT12, or ZT18 and euthanized 4 h following the EdU injection. iWAT and the eWAT were removed and immediately fractionated and processed as in the experimental designs 2 and 4.

#### Experimental design 6

Fig. 4, S4: Male C57BL/6J background mice were fasted for 18 h (fasting group), while the rest of mice were maintained on ad libitum feeding (control group). Animals then were injected at ZT0, ZT6, ZT12, or ZT18 with EdU or vehicle and euthanized at ZT4, ZT10, ZT16, or ZT20, respectively. iWAT and the eWAT were removed and immediately fractionated and processed as in the experimental designs 2, 4–5. During iWAT and eWAT fractionation into SVF, the MA were saved for further mRNA analysis. Mice were analyzed by infrared sensors to analyze diurnal and circadian home cage activity before and during the fasting paradigm (Fig. S4a).

#### Experimental design 7

Figs. 4: Male C57BL/6J mice were fed intermittently every 6 h (multiple-meal group), providing them the 100% of the total calorie intake in four equal aliquots of their total 24 h energy intake, while the rest of mice were maintained on ad libitum feeding (control group) for 48 h prior to EdU injections. Animals were injected at ZT0 or ZT12 with a single injection of EdU or vehicle and euthanized at ZT4 or ZT16, respectively. iWAT and the eWAT were removed and immediately fractionated and processed as in the experimental designs 2, 4–6.

#### Experimental design 8

Figs. 5 and S5: Male C57BL/6J mice were exposed to HFD starting at 6 weeks of age. After 6 weeks on the diet, one cohort of animals was provided VC instead of HFD for 2 weeks (Diet reversal group), with the remaining animals being maintained on HFD (control group). All mice were weighed weekly, and basal glucose was determined. Following the 2 weeks of diet reversal, mice were injected at ZT0 or ZT12 with a single injection of EdU or vehicle and tissues were harvested 4 h following injection (*n* = 4 per condition/ZT). iWAT and the eWAT were removed and processed as described in experimental designs 2, 4–7. MA were separated from SVF and saved for further mRNA analysis. Additional chunks for both WAT depots were stored for whole-tissue mRNA analysis and histology section processing and imaging as further detailed in the “Histology” section. Half of the animals were analyzed by infrared sensors to study diurnal and circadian activity before and during the diet reversal (Fig. 4c).

#### Experimental design 9

Figs. 6 and S6: Male *Clock*-deficient (*Clock*^*−/−*^) and WT littermate mice were exposed to VC and euthanized at approximately 26 weeks of age at ZT3, ZT9, ZT15, and ZT21 (*n* = 3 per genotype/ZT). iWAT and eWAT were removed and flash frozen in liquid nitrogen.

#### Experimental design 10

Fig. 6l: Male *Clock*-deficient (*Clock*^*−/−*^) and WT littermate mice (*n* = 5/genotype) were allowed to eat VC ad libitum for 14 weeks prior to injection of EdU or vehicle at ZT0. Following euthanasia, iWAT and the eWAT were quickly removed, weighed, and processed as described in experimental designs 2, 4–8. During iWAT and eWAT fractionation into SVF, total MA were prepared and counted using a hemocytometer (S6D). A separate age-matched cohort of animals (*n* = 3–4) were sacrificed at ZT4 to analyze whole-iWAT and -eWAT tissue, and for histology (Fig. 6f).

#### Experimental design 11

Figs. 7 and S7: Male *Clock*-deficient (*Clock*^*−/−*^) and WT littermate mice were fed VC and euthanized at 3 months of age at ZT4 (*n* = 3). iWAT and the eWAT were removed and immediately fractionated into SVF and MA. SVF were cultured as described in the “Cell culture” section.

### Human tissue

#### Experimental design 1

Fig. 8a–c: The clinical protocol associated with the study was approved by MD Anderson Institutional Review Board (IRB protocol HSC-IMM-07-0306). For each subject, the BMI (kg/m^2^) was calculated; obese was defined as BMI ≥ 30 (*n* = 6) while non-obese as BMI < 30 (*n* = 6). Select WAT specimens categorized according to BMI were from MD Anderson Cancer Center patients with clinically localized prostate cancer. No matching based on the clinical or pathological stage was performed. Periprostatic visceral (VIS) and abdominal subcutaneous (SC) WAT tissues were obtained at the time of prostatectomy. Informed consent was obtained from all subjects^73^.

#### Experimental design 2

Fig. 8d–g: The clinical protocol for this study was reviewed and approved by the Institutional Review Board at the McGovern Medical School, UT Health Science Center at Houston (IRB protocol HSC-MS-14-0514). Human abdominal subcutaneous (SC) and omental (VIS) fat tissue was acquired from obese bariatric surgery patients from the Memorial Hermann Hospital. Informed consent was obtained from all subjects. Excised tissues were either frozen for future processing of the whole tissue or alternatively, processed immediately for the generation of SVF, cultured or immediately stored at −80 °C for further analysis^74^.

### White adipose tissue fractionation

Freshly excised WAT was weighed, rinsed in PBS, washed in 1X Krebs–Ringer HEPES (Alfa Aesar, J67795) and minced in digestion buffer composed by 1 mg/ml type II collagenase (Sigma, C-6885) diluted in 1X Krebs–Ringer HEPES. The minced tissue was digested for 60–80 min at 37 °C and then filtered through a 100 μm × 70 μm nylon mesh sterile cell strainer (Fisher Scientific, 22363549 and 22363548, respectively) to remove undigested tissue and centrifuged at 500*g* for 5 min. The supernatant, containing MA, was aspirated and transferred to another tube while the pellet, containing the SVF, were immediately frozen at −80 °C or cultured as described in the “Cell culture” section. The MA were re-centrifuged at 500*g* for 5 min and the buffer was aspirated and discarded in order to isolate the MA, which were immediately frozen at −80 °C.

### Cell culture

Mouse SVF were washed with 1X PBS and centrifuged at 500*g* for 5 min to remove PBS. SVF were suspended in DMEM high-glucose media (HyClone, SH30243.01) supplemented with 10% fetal bovine serum (ATCC, 30-2020) and 100 U of penicillin/streptomycin/ neomycin (Fisher BP296150). The initial plating of primary cells was referred to as Passage 0. After 48–72 h of incubation at 37 °C and 5% carbon dioxide, cells were washed with 1X PBS and fed every other day until they achieved 75–90% confluence. Cells were passaged using trypsin 0.05% (Gibco, 25200-056) digestion after achieving a density of 75–90% and used directly in proliferation experiments (Cell division assay by EdU incubation) at Passage 3 or in serum shock experiments at Passage 5 in case of SVF isolated from *Clock*^*−/−*^ vs. WT mice.

### Cell division assay by EdU incubation

SVF from *Clock* KO and WT littermates at Passage 3 were incubated for 2 h with EdU (Cayman 20518), scraped, and then washed with 1X PBS. Cells were fixed in 4% formaldehyde (diluted in 1X PBS) and directly labeled with Click-iT EdU Alexa Fluor 647 kit (Invitrogen C10424) following manufacturer directions. Samples were analyzed by cell cytometry as described in the “Flow cytometry” section.

### Flow cytometry

The fluorescent signal generated by Click-iT^®^ EdU labeling was detected using logarithmic amplification of 633/635 nm excitation and a red emission filter; 50,000 events were counted using a low flow rate during acquisition. For adipose precursor cells sorting, SVF were incubated after EdU labeling, with CD34 (eFluor 450, eBioscience 48-0341-82, 1:20), CD31 (Alexa Fluor 488, BD 563607, 1:20), and CD45 (BUV395, BD 565967, 1:100) for 1 h in pre-sort buffer (BD, 563503) on ice and protected from light. Raw data were processed using FlowJo software (Tree Star, Ashland, OR, USA).

### Histology

Small chunks of iWAT and eWAT were fixed in formalin and embedded in paraffin as described in^75^. Adipose tissue sections (5 μM) were subsequently stained with hematoxylin and counterstained with eosin-phloxine solution. All images were acquired with a Cytation5 cell imager multi-mode reader (BioTek). Images captured at 20X magnification were analyzed with Adiposoft (ImageJ-Fiji) software to determine adipocyte size. Four fields per sample and three samples from each genotype were measured. Sizes were calculated from the average value of the cell area in all measured fields.

### Cytocentrifugation for SVF and APC

Purified SVF from approximately 400 mg tissue was resuspended in 600 μl of 1X PBS, 200 μl of which were loaded into each funnel. Loaded funnels were centrifuged for 5 min at approximately 70*g*, using a Cytospin4^®^. To prepare the slides for APC, purified APCs from 400 mg tissue were resuspended in 200 μl and the full amount was deposited on a slide.

### Immunofluorescence

Slides containing cytospin-isolated cells were fixed and permeabilized with 3.7% formaldehyde and 0.1% triton for 20 min at room temperature (RT). After blocking with 5% BSA in PBS for 1 h, slides were incubated with anti-Ki67 (1:200, thermofisher-RM-9106), CD45 (1:200, Invitrogen-16-0451), and endomucin antibodies (1:200, R&D-AF4666) overnight at 4 °C. Slides were washed with PBST buffer followed by incubation with donkey anti-rat (1:500, Invitrogen A-21208), donkey anti-goat secondary (1:500, Invitrogen A-11055) or antibodies conjugated with Alexa Fluor 488 or donkey anti-rabbit IgG conjugated with Alexa Fluor 647 (1:500, Invitrogen A31573) in a dark chamber for 1 h at RT. Nuclei were stained by 4,6-diamidino-2-phenylindole (DAPI). Images were captured using Nikon Fluorescent microscope.

### Circadian activity

Unless otherwise indicated, mice were singly housed in cages containing infrared sensors (STARR Life Sciences, PA) to monitor total home cage diurnal activity. Data were collected using VitalView (STARR Life Sciences) and locomotion data were analyzed using ClockLabs software (ActiMetrics, IL).

### Metabolic phenotyping

Mice were placed at RT (22–24 °C) in metabolic cages (Comprehensive Lab Animal Monitoring System-CLAMS; Columbus Instruments) and VC or HFD was replaced with ground versions of the same diets immediately upon recording. Food and water were provided ad libitum. Food intake data were collected and averaged over the course of 4 days following a 2-day habituation period to the chambers. Data were analyzed using Oxymax V 4.87.

### Protein extraction

For whole-cell lysates, epididymal fat tissue was homogenized in RIPA as described in^67^. Details are provided in Supplemental Experimental Procedures.

### Western blotting

Immunoblotting for epididymal adipose tissue was performed as described in^67^. Details are provided in Supplemental Experimental Procedures.

### RNA extraction and reverse transcriptase, quantitative PCR

RNA extraction, reverse transcriptase and quantitative PCR was performed as described in^67^. Details are provided in Supplemental Experimental Procedures. All primer sequences can be found in Table S2.

### Third party images

Third party images were used from the Library of Science and Medical Illustrations, at http://www.somersault1824.com/resources/. A link to the license address is: https://creativecommons.org/licenses/by-nc-sa/4.0/.

### Statistical analysis

Data are shown as mean with SEM. Significance (*p* < 0.05) was determined by two-tailed unpaired Student’s *t*-test (*n* > 10) or by the non-parametric Mann–Whitney *U* test (*n* < 10). For experiments involving more than one variable, significance was assessed using two-way ANOVA followed by Tukey’s or Sidak’s post hoc test, as indicated in the figure legends. When data were analyzed by both two-way ANOVA and Mann–Whitney or Student’s *t*-test, results for Student’s *t*-test are displayed in red. Data were analyzed using GraphPad Prism 7. Data points that exceeded a predetermined 1.5 standard deviation from the mean were considered outliers. For experiments assessing rhythmicity, we used both the JTK_Cycle non-parametric test^28^ and cosinor analysis. Individual biological replicates for each time point were used for the analysis. JTK_Cycle (R Studio) was applied, using a window of 20–28 h to capture circadian oscillations. For serum shock experiments, rhythmicity was ascertained from all values excluding the ZT0 (unsynchronized) time point. To calculate if variables fit to the cosinor model, we performed cosinor regression in R studio with the packages “cosinor” to calculate rhythm *p*-value, mesor and amplitude and “cosinor2” to calculate the acrophase in radians, which also was converted into zeitgeber time. Generally, random assignment was used in animal experiments except when placing them into initial diet groups, where non-randomization was chosen to ensure that each group was weight-matched prior to a switch to HFD. When possible, experimental evaluation was performed blind to the experimental conditions (i.e., specifically for western blot quantification, image capture and analysis of H&E staining, and cell cytometry analysis).

### Reporting summary

Further information on research design is available in the Nature Research Reporting Summary linked to this article.

## Supplementary information

Supplementary information file.

Supplementary data.

Reporting summary.

## Data Availability

All relevant data to this manuscript are available from the authors. All data underlying figures can be found in the Source Data file.
