# Publisher Correction: Cellular and physiological circadian mechanisms drive diurnal cell proliferation and expansion of white adipose tissue

**DOI:** 10.1038/s41467-021-24864-5

**Published:** 2021-07-20

**Authors:** Aleix Ribas-Latre, Rafael Bravo Santos, Baharan Fekry, Yomna M. Tamim, Samay Shivshankar, Alaa M. T. Mohamed, Corrine Baumgartner, Christopher Kwok, Claudia Gebhardt, Angielyn Rivera, Zhanguo Gao, Kai Sun, John T. Heiker, Brad E. Snyder, Mikhail G. Kolonin, Kristin L. Eckel-Mahan

**Affiliations:** 1 Institute of Molecular Medicine, McGovern Medical School at the University of Texas Health Science Center, Houston, TX 77030 USA; 2 Helmholtz Institute for Metabolic, Obesity and Vascular Research (HI-MAG) of the Helmholtz Zentrum München at the University of Leipzig and University Hospital Leipzig, 04103 Leipzig, Germany; 3 Faculty of Medicine, Ain Shams University, Cairo, 1181 Egypt; 4 Memorial Hermann Texas Medical Center, Houston, TX 77030 USA; 5 Department of Integrative Biology and Pharmacology, McGovern Medical School at the University of Texas Health Science Center, Houston, TX 77030 USA

**Keywords:** Fat metabolism, Feeding behaviour, Obesity, Metabolic diseases, Type 2 diabetes

Correction to: *Nature Communications* 10.1038/s41467-021-23770-0, published online 9 June 2021.

The original version of this Article contained an error in Ref. 72, which was incorrectly given with the wrong author names as: Mendez-Ferrer, S., Lucas, D., Battista, M. & Frenette, P. S. Age-associated telomere attrition in adipocyte progenitors predisposes to metabolic disease. *Nat. Metab*. **2**, 1482–1497 (2020). The correct form of Ref. 72 is: Gao, Z. et al. Age-associated telomere attrition in adipocyte progenitors predisposes to metabolic disease. *Nat. Metab*. **2**, 1482–1497 (2020). This has been corrected in the PDF and HTML versions of the Article.

