# Supplementary material for: Cellular and physiological circadian mechanisms drive diurnal cell proliferation and expansion of white adipose tissue
Source: Nat Commun. 2021 Jun 9;12:3482. doi: 10.1038/s41467-021-23770-0 (PMC8190103; doi:10.1038/s41467-021-23770-0)
Supplement: Supplementary file 1 — Supplementary information file. [file 41467_2021_23770_MOESM1_ESM.pdf]

Cellular and Physiological Circadian Mechanisms Drive Diurnal Cell Proliferation and Expansion of White Adipose Tissue

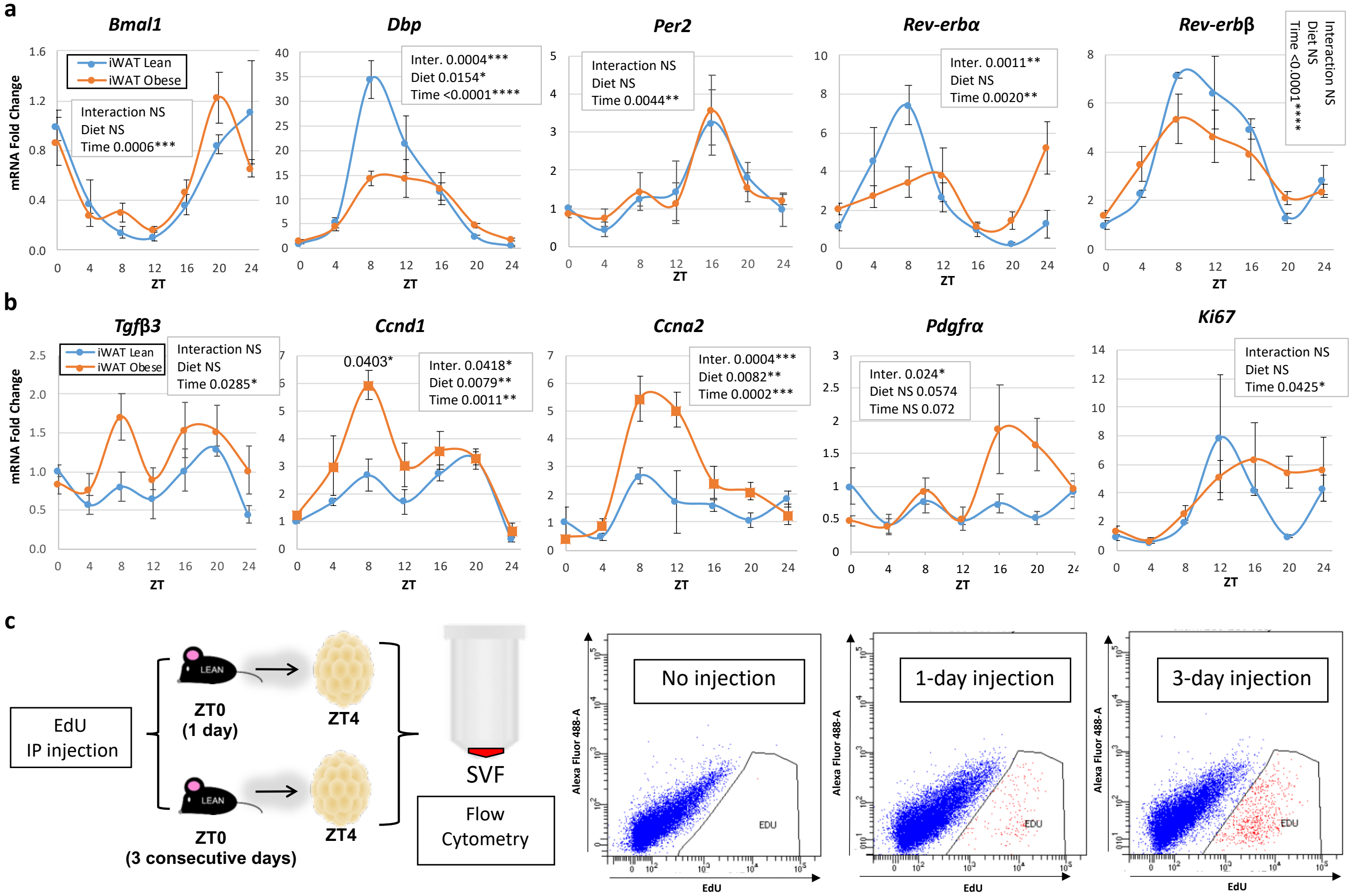

Figure S1. Sourcing the cellular identity of proliferating stromal vascular cells in adipose tissue (a-b) RT-PCR analysis reveals expression of circadian **(a)** and proliferation **(b)** genes in the inguinal adipose tissue (iWAT) from lean (blue) and obese (orange) mice throughout the circadian cycle (*n*=4 animals/*Zeitgeber* time, ZT). mRNA levels for lean mice at ZT0 were set to 1 for all genes. **(c)** Model to determine suitable EdU injection (left panel) and representative fluorescence activated cell sorting of EdU-positive cells from epididymal (eWAT) stromal vascular cells (SVF) isolated at ZT4 after control injection, a single 1-day EdU injection or a single EdU injection on three consecutive days (right panel). Data are represented as mean ± SEM. *p < 0.05, **p < 0.01, ***p < 0.001, ****p < 0.0001. Significance (p < 0.05) determined by two-way ANOVA and Sidak's post-hoc test in **a-b**. (Related to Figure 1.)

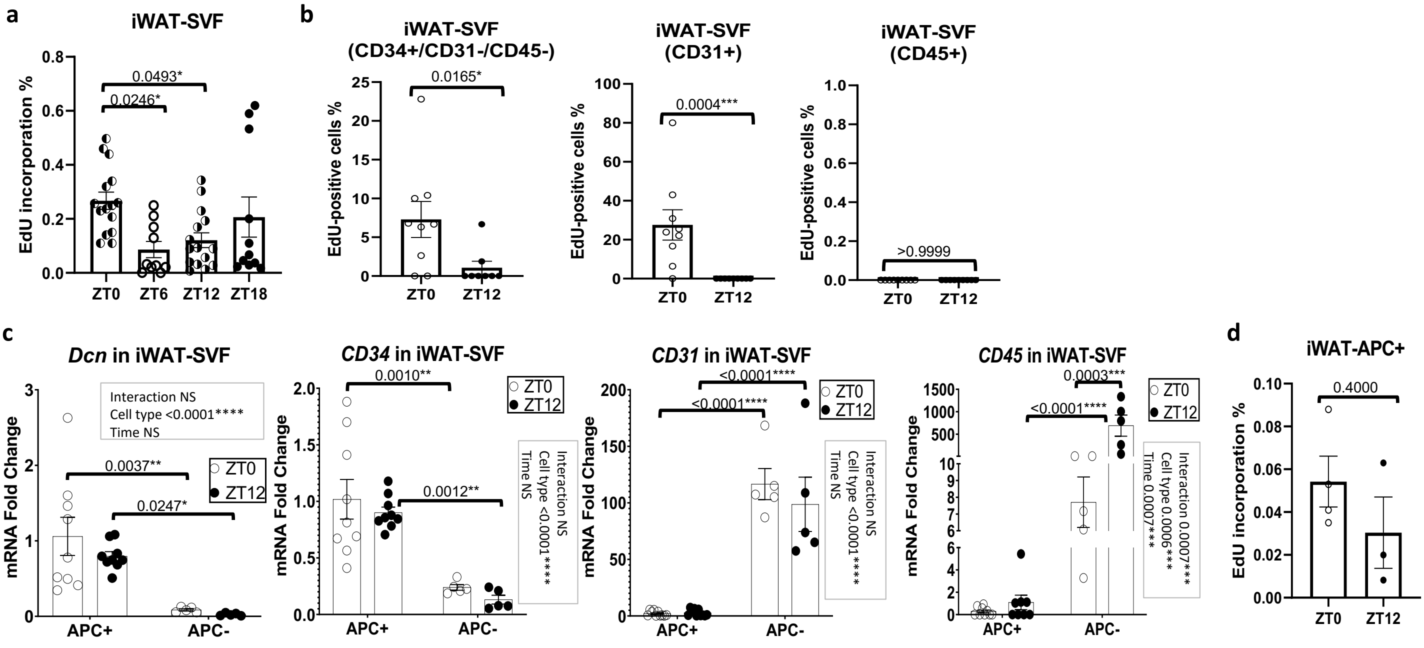

Figure S2. Diurnal variation in the proliferation of inguinal adipose tissue stromal vascular cells *in vivo* (a) Quantification of fluorescence activated cell sorting of EdU-positive cells from iWAT-SVF isolated four hours following EdU injections at *Zeitgeber* time (ZT)0, ZT6, ZT12 and ZT18 (*n*=17 animals/ZT0 and ZT12 and *n*=12 animals/ZT6 and ZT18). **(b)** Quantification of fluorescence activated cell sorting of total $EdU^+/CD34^+/CD31^-/CD45^-$ (left panel), $EdU^+/CD31^+$ (middle panel) and $EdU^+/CD45^+$ (right panel) SVF from iWAT isolated at ZT4 and ZT16, four hours following a single EdU injection (*n*=10 animals/ZT). **(c)** RT-PCR analysis reveals expression of progenitor (Dcn and CD34), endothelial (CD31) and lymphocyte (CD45) markers in iWAT/adipose progenitor cells (APC) and iWAT/non-APC cells from lean mice isolated at ZT4 and ZT16 (*n*=5 animals for APC- and *n*=10 animals for APC+/ZT). mRNA levels for lean mice at APC+/ZT0 were set to 1 for all genes. **(d)** Quantification of EdU-positive cell sorting from iWAT-APC isolated at ZT4 and ZT16, four hours following a single EdU injection (*n*=5 animals/ZT). **B-d**, white and black circles represent ZT0 and ZT12 respectively. Data are represented as mean ± SEM. *p < 0.05, **p < 0.01, ***p < 0.001, ****p < 0.0001. Significance (p < 0.05) determined by one-way ANOVA followed by Tukey's post-hoc test in **a**; two-tailed Mann–Whitney U test in **b** and **d** and two-way ANOVA and Tukey's post-hoc test in **c**. (Related to Figure 2.)

S3

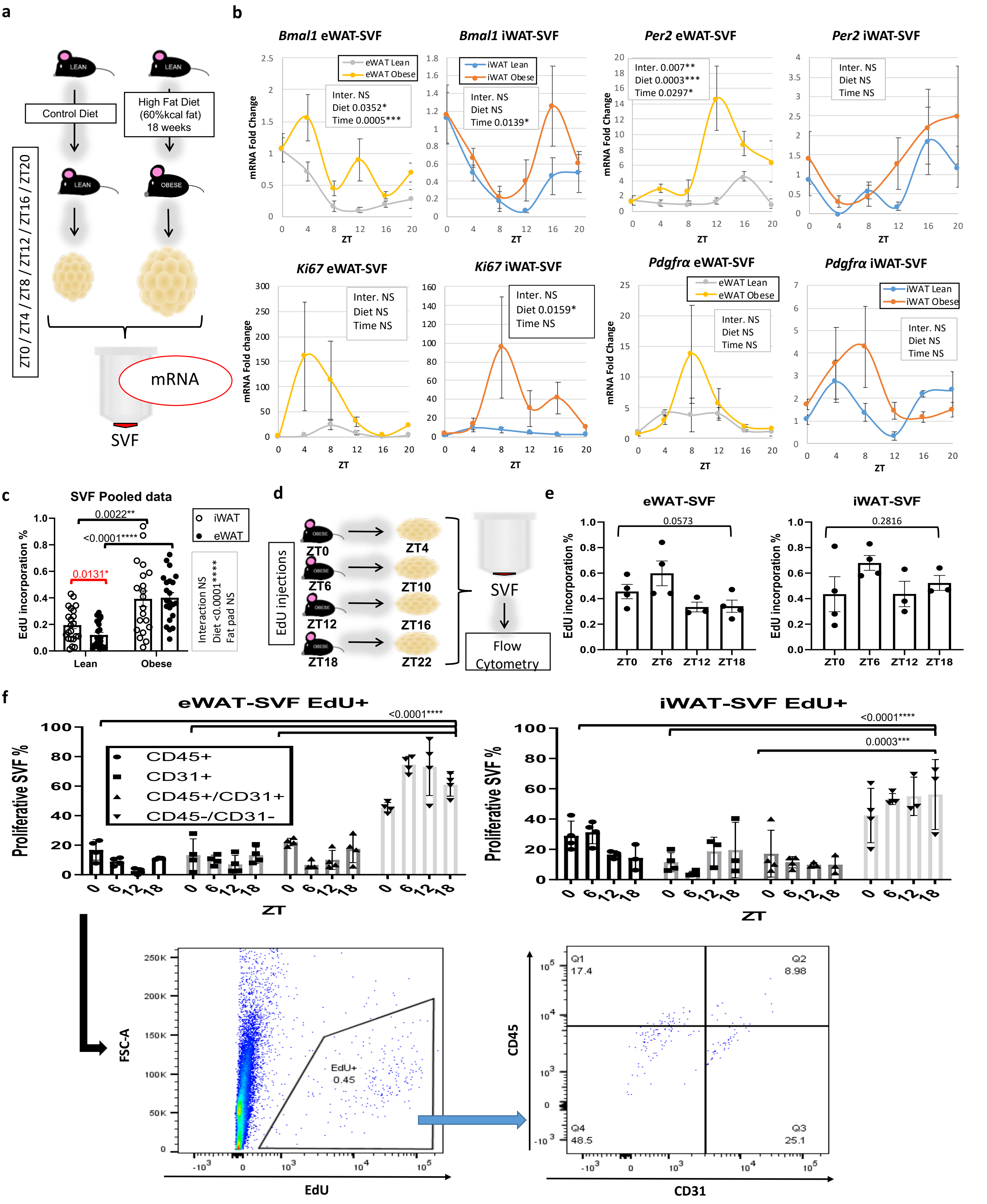

a
b
Control Diet
High Fat Diet (60%kcal fat) 18 weeks
LEAN
OBESE
ZT0 / ZT4 / ZT8 / ZT12 / ZT16 / ZT20
mRNA
SVF
Bmal1 eWAT-SVF
Bmal1 iWAT-SVF
Per2 eWAT-SVF
Per2 iWAT-SVF
eWAT Lean
eWAT Obese
iWAT Lean
iWAT Obese
Inter. NS
Diet 0.0352*
Time 0.0005***
Inter. NS
Diet NS
Time 0.0139*
Inter. 0.007**
Diet 0.0003***
Time 0.0297*
Inter. NS
Diet NS
Time NS
mRNA Fold Change
ZT
Ki67 eWAT-SVF
Ki67 iWAT-SVF
Pdgfrα eWAT-SVF
Pdgfrα iWAT-SVF
Inter. NS
Diet NS
Time NS
Inter. NS
Diet 0.0159*
Time NS
mRNA Fold change
c
SVF Pooled data
0.0022**
<0.0001****
0.0131*
iWAT
eWAT
EdU incorporation %
Lean
Obese
Interaction NS
Diet <0.0001****
Fat pad NS
d
EdU injections
ZT0
ZT4
ZT6
ZT10
ZT12
ZT16
ZT18
ZT22
SVF
Flow Cytometry
e
eWAT-SVF
iWAT-SVF
0.0573
0.2816
ZT0
ZT6
ZT12
ZT18
f
eWAT-SVF EdU+
iWAT-SVF EdU+
<0.0001****
0.0003***
CD45+
CD31+
CD45+/CD31+
CD45-/CD31-
Proliferative SVF %
ZT
FSC-A
EdU
EdU+
0.45
CD45
CD31
Q1
17.4
Q2
8.98
Q4
48.5
Q3
25.1

Figure S3 Diurnal regulation of stromal vascular cell proliferation in lean vs. obese mice (a) Model of diet-induced obesity: epididymal (eWAT) and inguinal (iWAT) stromal vascular cells (SVF) were isolated from tissue harvested every four hours throughout the circadian cycle (*n*=4 animals/*Zeitgeber* time, ZT) for further analysis. **(b)** RT-PCR analysis reveals expression of *Bmal1, Per2, Ki67* and *Pdgfrα* in the eWAT (left panels) and iWAT (right panels) SVF from obese (yellow line for eWAT and orange line for iWAT) and lean (gray line for eWAT and blue line for iWAT) mice throughout the 24-h cycle (*n*=4 animals/ZT). mRNA levels for lean mice at ZT0 were set to 1. **(c)** Quantification of EdU-positive cells following flow cytometry of both iWAT (white circles) and eWAT SVF (black circles) from lean and obese mice pooling data from ZT0 and ZT12 (*n*=24 animals/diet) (related to Figure 2H-I). **(d)** Model to determine diurnal proliferation in obese mice: 5-Ethynyl-2'-deoxyuridine (EdU) was administered to obese mice, fed *ad libitum* HF diet, at ZT0, ZT6, ZT12 and ZT18. eWAT and iWAT SVF were isolated from fat pads harvested four hours following EdU injection (*n*=4 animals/ZT). **(e)** Quantification of EdU-positive cells following flow cytometry of eWAT (left panel) and iWAT (right panel) SVF isolated four hours after single EdU injections at ZT0, ZT6, ZT12 or ZT18 (*n*=4 animals/ZT). **(f)** Quantification of $CD45^+$ (represented by circles), $CD31^+$ (represented by squares), $CD45^+/CD31^+$ (represented by triangles facing up) and $CD45^-/CD31^-$ (represented by triangles facing down) EdU-positive cells following flow cytometry of eWAT (left panel) and iWAT (right panel) SVF harvested four hours following single EdU injections at ZT0, ZT6, ZT12 or ZT18 to obese mice (*n*=4 animals/ZT). Representative fluorescence activated cell sorting (lower panel). Data are represented as mean ± SEM. *p < 0.05, **p < 0.01, ***p < 0.001, ****p < 0.0001. Significance (p < 0.05) determined by two-way ANOVA followed by Sidak's post-hoc test in **b** and Tukey's post-hoc test in **c**; two-tailed, unpaired T-test for red asterisks and one-way ANOVA followed by Tukey's post-hoc test in **e-f**. (Related to Figure 3.)

S4

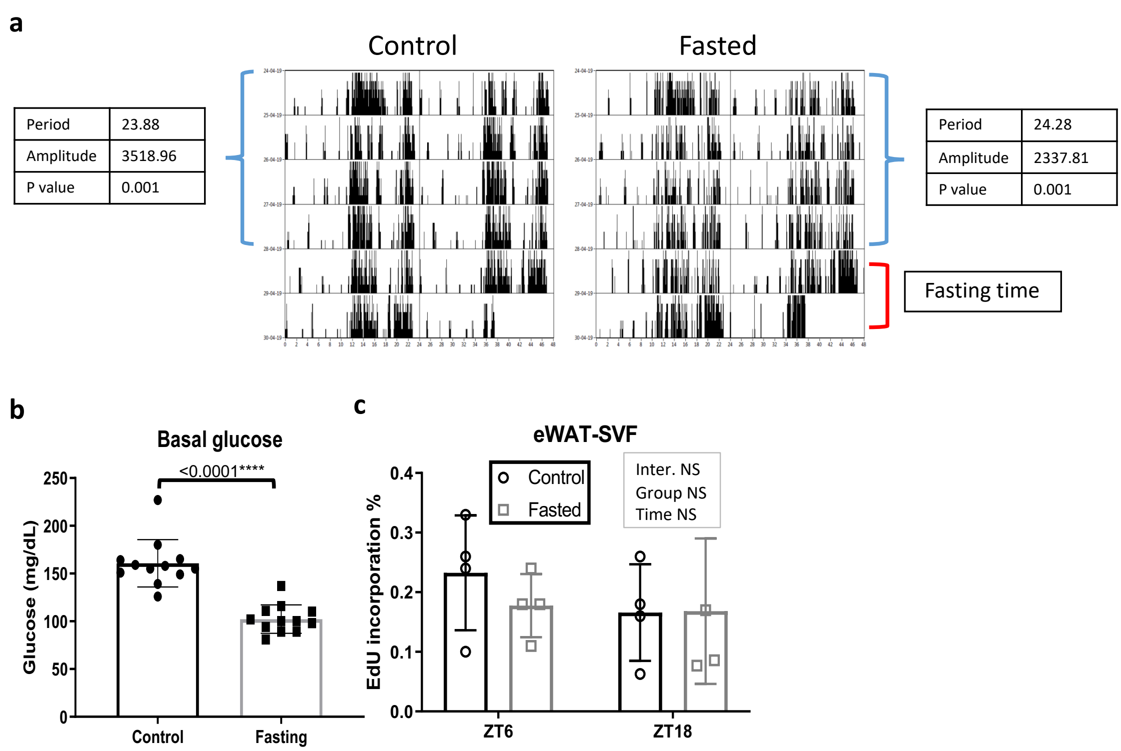

Figure S4 Fasting-induced disruption of diurnal stromal vascular cell (SVF) proliferation *in vivo* (a) Diurnal locomotion of control (*ad libitum*-fed) and fasted mice as measured by infrared sensors tracking home cage activity. **(b)** Basal glucose of control and fasted mice (*n*=6 animals/feeding condition). **(c)** Quantification of fluorescence activated cell sorting of EdU-positive SVF-eWAT isolated from lean mice fed *ad libitum* control chow or fasted for 18 hours. Mice were subjected to 5-Ethynyl-2'-deoxyuridine (EdU) injection at ZT6 and ZT18 and sacrificed four hours following EdU injection (*n*=4 animals/*Zeitgeber* time, ZT and feeding condition). **B-c**, circles and squares represent fed *ad libitum* and fasted mice, respectively. Data are represented as mean ± SEM. *p < 0.05, **p < 0.01, ***p < 0.001, ****p < 0.0001. Significance (p < 0.05) determined by 2-tailed unpaired T-test in **b** and two-way ANOVA and Tukey's post-hoc test in **c**. (Related to Figure 4.)

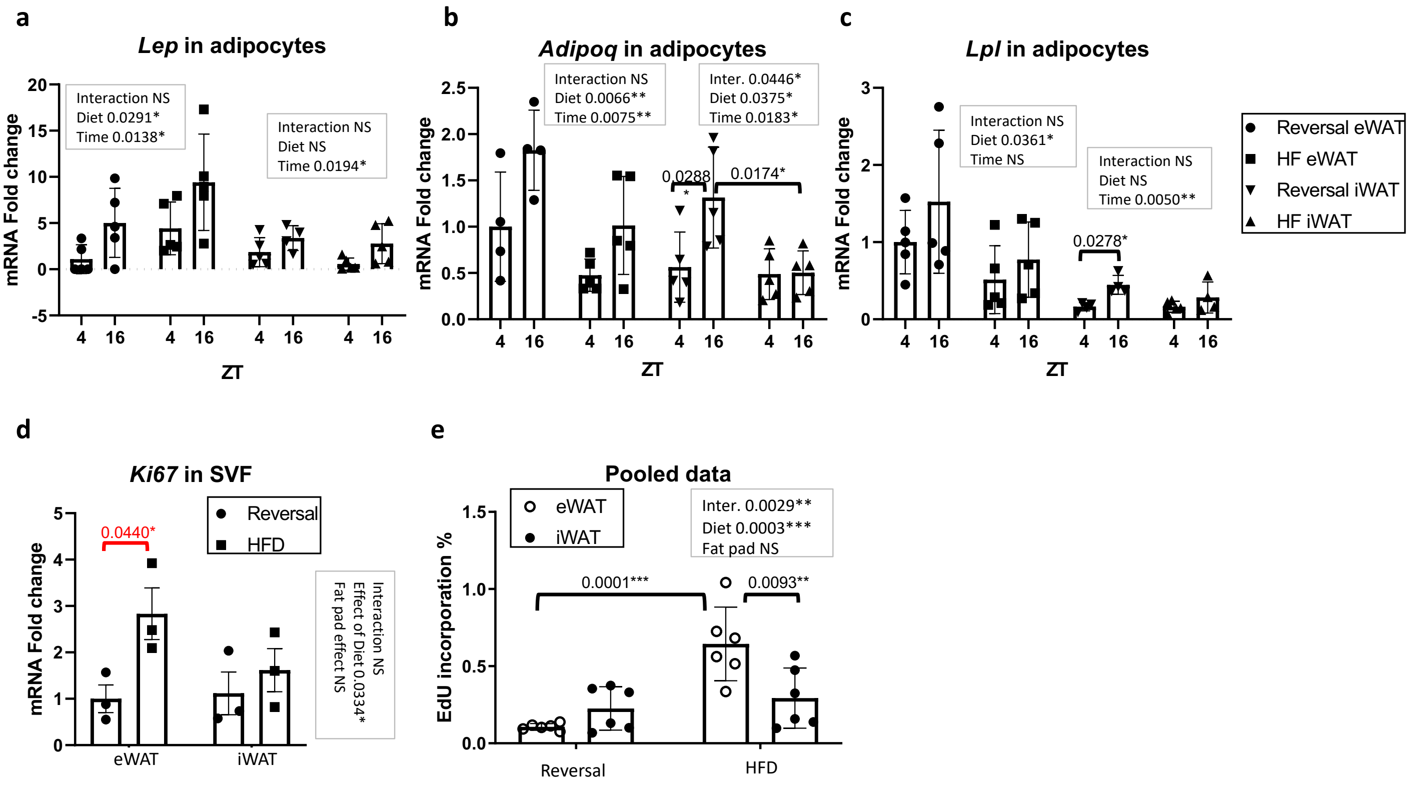

Figure S5 Diurnal patterns of adipocyte gene expression and proliferating immune cells in high fat (HF) diet-fed and diet reversal mice (a-c) qPCR reveals expression of leptin (*Lep*) **(a)**, adiponectin (*Adipoq*) **(b)** and lipoprotein lipase (*Lpl*) **(c)** in pure adipocytes from epididymal (eWAT) and inguinal (iWAT) with or without diet reversal at ZT4 and ZT16 (*n*=5 animals/diet and *Zeitgeber* time, ZT). mRNA levels from eWAT collected at ZT4 for mice subjected to diet reversal were set to 1. **(d)** qPCR reveals expression of *Ki67* in both eWAT and iWAT SVF with or without diet reversal at ZT4 (*n*=3 animals/diet). mRNA levels from the eWAT SVF for mice subjected to diet reversal were set to 1. For all bar graphs **(a-d)**, black circles and squares represent mice fed chow diet reversal or HFD respectively (triangles facing down or up respectively for iWAT in **a-c**). **(e)** Quantificaiton of pooled data of EdU-positive cells following flow cytometry of eWAT (white circles) and iWAT SVF (black circles) from mice with or without diet reversal regardless of circadian time (*n*=8 animals/diet). Data are represented as mean ± SEM. *p < 0.05, **p < 0.01, ***p < 0.001, ****p < 0.0001. Significance (p < 0.05) determined by two-way ANOVA followed by Tukey's post-hoc test in **a-e**. (Related to Figure 5.)

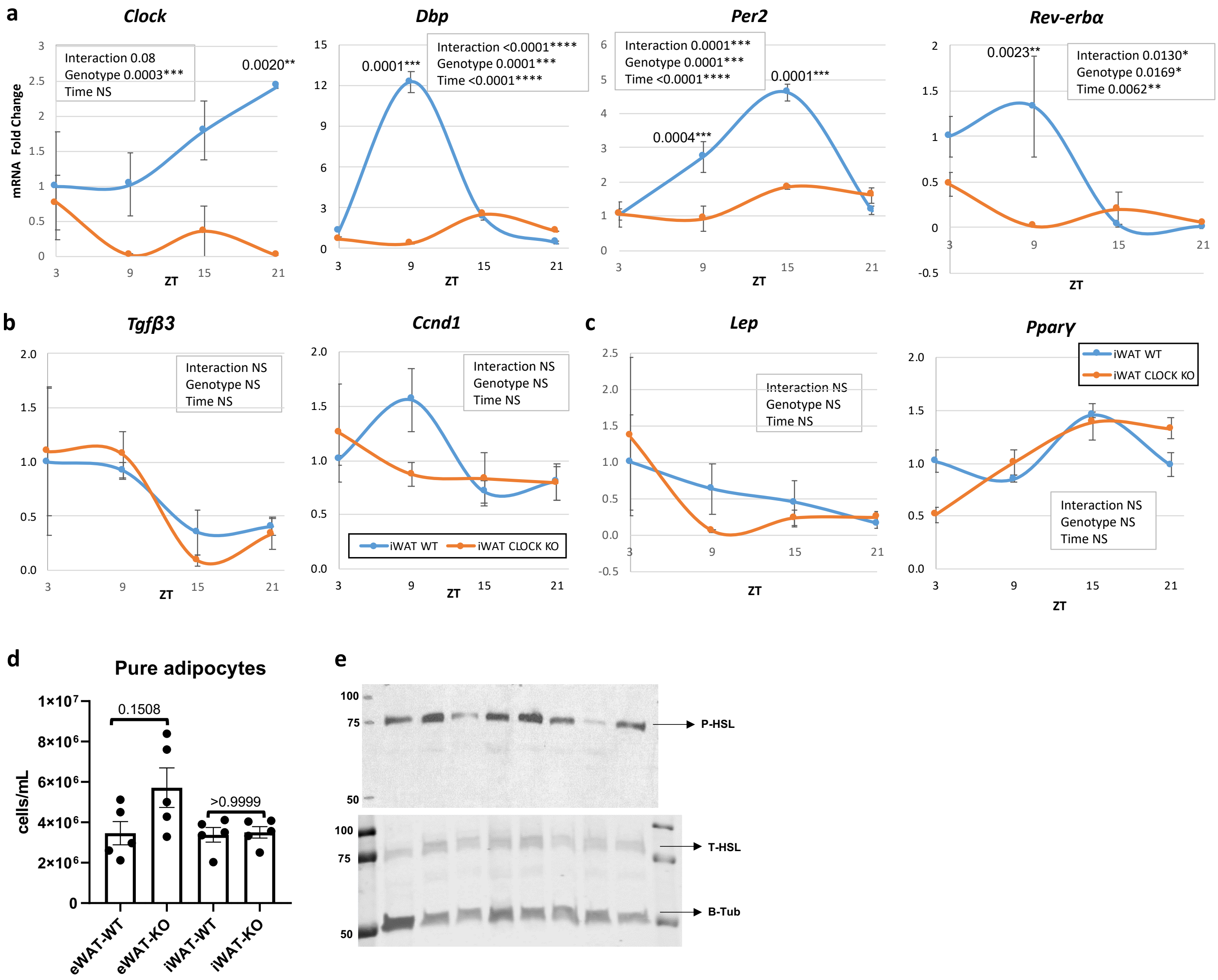

Figure S6 Analysis of inguinal (iWAT) gene expression in *Clock* KO vs. WT mice (a-c) RT-PCR analysis reveals expression of circadian clock **(a)**, proliferation **(b)** and adipogenesis-related genes **(c)** in the iWAT from *Clock* KO (orange line) and WT littermate (blue) mice throughout the diurnal cycle (n=3 animals/ *Zeitgeber* time, ZT) . mRNA levels for WT mice at ZT3 were set to 1. **(d)** Number of pure adipocytes in eWAT and iWAT from *Clock* KO and WT littermate mice (n=5 animals/genotype). **(e)** Representative blots with pooled samples reveals expression of phosphorylated hormone sensitive lipase (P-HLS), total HSL (T-HLS) and β-Tubulin throughout the circadian cycle (n=3 animals pooled/ZT) in eWAT. The same nitrocellulose membrane was used to incubate all three antibodies while for imaging P-HLS was detected in a film developer and the others in a CLx imaging system as shown in supplemental experimental procedures. Data are represented as mean ± SEM. *p < 0.05, **p < 0.01, ***p < 0.001, ****p < 0.0001. Significance (p < 0.05) determined by two-way ANOVA followed by Sidak's post-hoc test in **a-c** and two-tailed Mann–Whitney U test in **d**. (Related to Figure 6.)

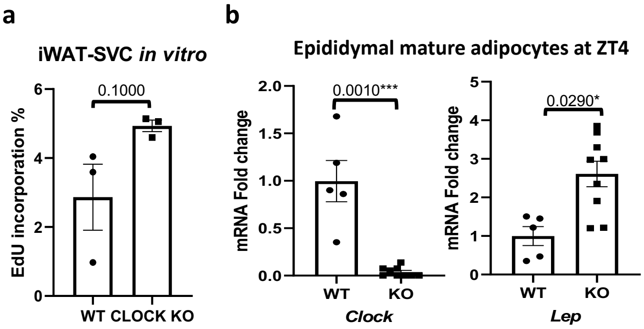

Figure S7 Proliferative capacity of cultured inguinal stromal vascular cells (SVF) of *Clock* KO vs. wild-type littermate mice (a) Quantification of *in vitro* fluorescence activated cell sorting of EdU-positive cells from iWAT SVF isolated from *Clock* KO vs. WT littermates (*n*=3 animals/genotype). **(b)** RT-PCR analysis reveals expression of *Clock* (left panel) and *Lep* (right panel) in pure adipocytes of epidigymal (eWAT) from *Clock* KO (*n*=9) vs. WT littermates (*n*=5). mRNA levels for WT mice were set to 1. Data are represented as mean ± SEM. *p < 0.05, **p < 0.01, ***p < 0.001, ****p < 0.0001. Significance (p < 0.05) determined by two-tailed Mann–Whitney U test **(a-b)**. For all bar graphs, circles and squares represent WT or CLOCK KO mice respectively. (Related to Figure 7.)

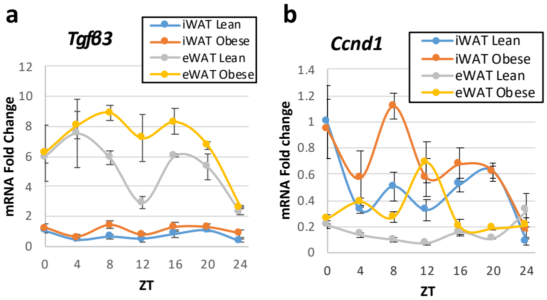

Figure S8 *Tgfβ3* and *Ccnd1* expression in lean and obese mouse adipose tissue depots (a) RT-PCR analysis reveals expression of *Tgfβ3* in epidydimal fat (eWAT) and inguinal fat (iWAT) from obese and lean mice throughout the circadian cycle (*n*=4 mice/ZT). mRNA levels from lean mouse ZT0 iWAT set to 1. **(b)** RT-PCR analysis reveals expression of *Ccnd1* in eWAT (gray and yellow lines) and iWAT (blue and orange lines) of obese (yellow and orange lines) and lean (gray and blue lines) mice throughout the circadian cycle (*n*=4 mice/ZT). mRNA levels from iWAT for lean mice at ZT0 were set to 1.

S9

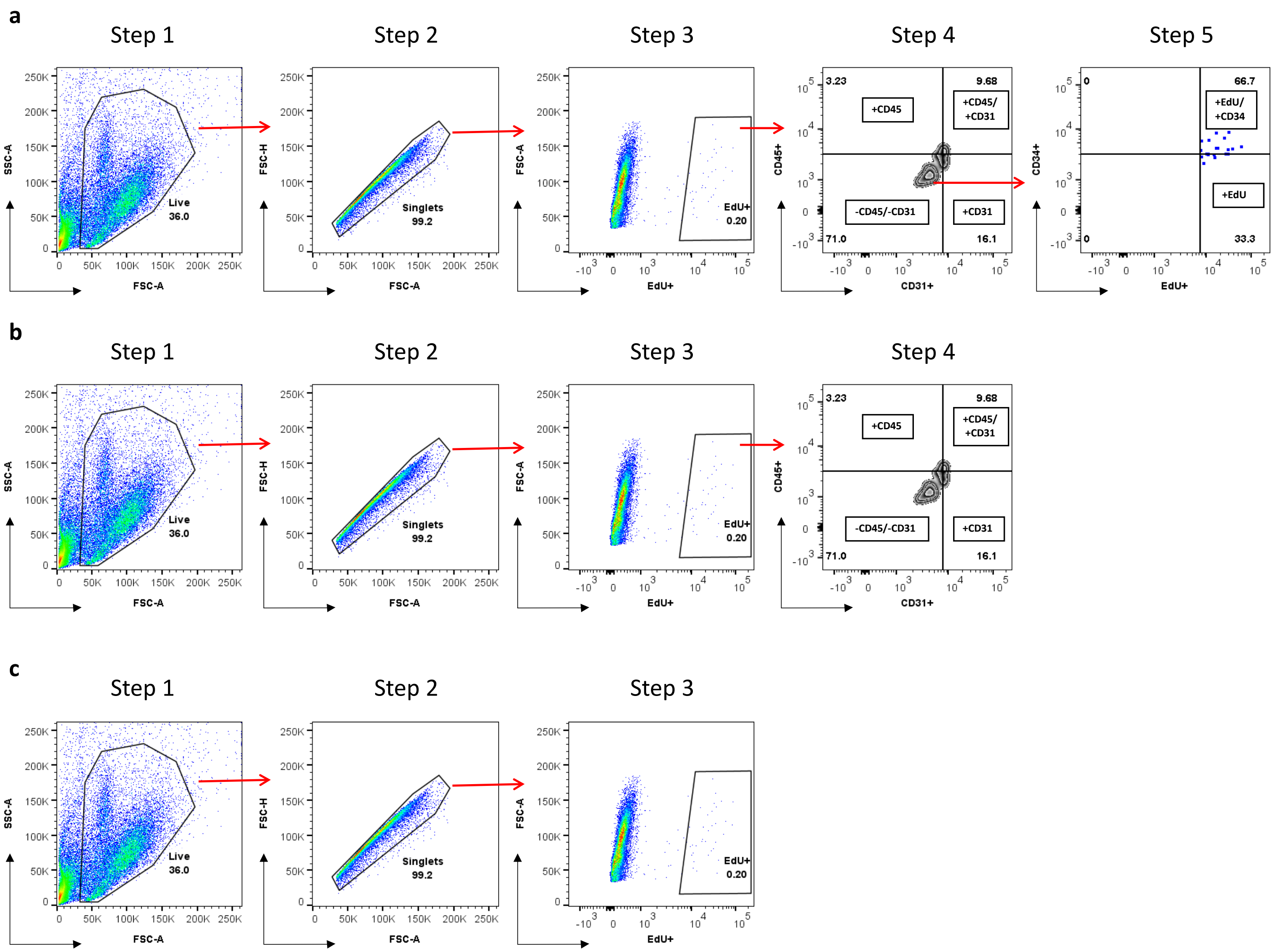

Figure S9 Gating strategies used for cell sorting and quantification (a) Representative fluorescence activated cell sorting strategy of EdU and endothelial (CD31), hematopoietic (CD45) and adipocyte progenitor (CD34) markers to identify proliferative adipocyte progenitors ($CD34^+/CD31^-/CD45^-$) from lean mice at different times of the day as presented on Fig.1g and S2b. **(b)** Representative fluorescence activated cell sorting strategy to identify proliferative endothelial (CD31) and hematopoietic (CD45) cells from obese mice at different times of the day as presented on S.3f. **(c)** Representative fluorescence activated cell sorting strategy to quantifiy EdU positive cells as presented on Fig.1f, Fig.2h, Fig.3e-f, Fig.4e, Fig.5h-i, Fig.6i, Fig. 7b, S1c, S2a, S3c-e, S4c, S5e and S7a.

Supplemental Experimental Procedures

Protein extraction For whole cell lysates, eWAT tissue was homogenized in RIPA lysis buffer (50 mM Tris, 150 mM NaCl, EDTA 5mM, $MgCl_2$ 15mM and NP-40 1%) for 15 s using a MagNA Lyser (Roche, IN, US). Samples were nutated for 15 min and spun at high speed for 15 min to eliminate insoluble material.

Western blotting Protein concentration was determined by using Pierce BCA Protein Assay Kit (Thermo Scientific, 23225) according to manufacturer directions. Lysates were diluted with RIPA and 5X Laemmli (0.5M Tris-HCl, pH 6.8; 10% glycerol; 2% (w/v) SDS; 5% (v/v) β-mercaptoethanol; and 0.05% bromophenol blue). 40 µg of total protein were loaded and separated on an 8% SDS-polyacrylamide gel and transferred to nitrocellulose (Bio-Rad, 162-0115). The membranes were incubated overnight at 4 °C with primary monoclonal antibodies diluted 1:1,000 and directed against Tubulin (Abcam, ab6046), T-HSL (Cell Signaling, 4107S) and P-HSL (Cell Signaling, 4126). After washing with TBS-Tween, the blots were incubated at a 1:15,000 dilution at room temperature for 1h with a secondary anti-rabbit antibody conjugated to horseradish peroxidase (HRP) (BioRad, 172-1019) or IR Dye 800CW donkey anti-rabbit (Li-Cor, 926-32213, NE, USA). Immunoreactive proteins were visualized with an enhanced chemiluminescence substrate kit (ECL plus; Bio-Rad, 102030812, CA, US) according to the manufacturer's instructions. Images were obtained on a film developer (AFP Imaging) using HyBlot autoradiography film (E3012, Denville Scientific INC) or with a CLx imaging system (Odyssey classic; Li-Cor, 004-1354, NE, USA) for fluorescent proteins. Band quantification was performed with ImageJ software (NIH, USA). The results were expressed as relative intensity.

RNA extraction and reverse transcriptase, quantitative PCR Total RNA was isolated from cells and liver using TRIzol reagent (Fisher Scientific, 15-596-018) according to the

manufacturer's protocol. One microgram of total RNA was used for cDNA synthesis using an iScript cDNA synthesis kit (Bio-Rad, 170-8891). Advanced Universal SYBR Green Super mix (Bio-Rad, 1725275) was used for qPCR amplification using a Bio-Rad C1000. PCR protocol settings were as follows: 95 °C for 30 s, 95 °C for 10 s, 62 °C for 30 s, and then 39 cycles at 65 °C for 31 s and 65 °C for 5 s. Ribosomal subunit 18 s expression was used as control. The fold change in mRNA expression for each gene was calculated using $2^{-\Delta\Delta C}$.

KEY RESOURCES TABLE

REAGENT or RESOURCE	SOURCE	IDENTIFIER
**Antibodies**		
Rabbit B-TUBULIN (1:1000)	Abcam	Cat#ab6046
Rabbit P-HSL (Ser660) (1:1000)	Cell Signaling	Cat#4126
Rabbit T-HSL (1:1000)	Cell Signaling	Cat#4107S
(H=L)-HRP conjugate monoclonal anti-rabbit (1:15000)	Bio-Rad	Cat#172-1019
IR Dye 800CW donkey anti-rabbit (1:15000)	Li-Cor	Cat#926-32213
BUV395 Rat Anti-Mouse CD45 (1:100)	bdbiosciences	Cat#565967
Alexa Fluor 488 Rat Anti-Mouse CD31 (1:20)	bdbiosciences	Cat#563607
eFluor 450 Rat Anti-Mouse CD34 (1:20)	eBioscience	Cat#48-0341-82
Ki67 (1:200) (cytospin)	R&D	AF4666
CD45 (1:200) (cytospin)	Invitrogen	16-0451
Endomucin (1:200) (cytospin)	R&D	AF4666

donkey anti rat (1:500) (cytospin)	Invitrogen	A-21208
donkey anti goat (1:500) (cytospin)	Invitrogen	A-11055
donkey-anti-rabbit IgG (1:500) (cytospin)	Invitrogen	A31573
**Biological Samples**		
Human and mouse subcutaneous and visceral adipose tissue	This paper	
**Chemicals, Peptides, and Recombinant Proteins**		
5'Ethynyl-2'-deoxyuridine (EdU)	Cayman	Cat#20518
Type II Collagenase	Sigma	Cat# C-6885
Penicillin/streptomycin/neomycin	Fisher	Cat#BP296150
Trypsin 0.05%	Gibco	Cat#25200-056
**Critical Commercial Assays**		
Click-iT EdU Alexa Fluor 647 kit	Invitrogen	Cat# C10424
Pierce BCA Protein Assay Kit	Thermo Scientific	Cat#23225
ECL plus (chemiluminescence substrate kit)	Bio-Rad	Cat#102030812
iScript cDNA synthesis kit	Bio-Rad	Cat#170-8891
Progenitor isolation kit	Miltenyi Biotec	Cat#5200501358
**Experimental Models: Cell Lines**		
Mouse and human primary cultured stromal vascular cells	This paper	
**Experimental Models: Organisms/Strains**		
Mouse: C57BL/6J	Jackson Laboratory	JAX: 000664

Mouse: *Clock*-deficient (*Clock*$^{-/-}$)	Gifted from David Weaver	33
**Software and Algorithms**		
ClockLabs	ActiMetrics	
ImageJ	NIH	https://imagej.nih.gov/ij/download.html
Prism 7	Graphpad	https://www.graphpad.com/scientific-software/prism/
R Studio (JTK_Cycle and cosinor)		
FlowJ		http://docs.flowjo.com/d2/
**Other**		
PicoLab Rodent Diet 20 EXT IRR (13% Kcal from fat)	LabDiet	Cat#5R53
Rodent Diet with 60% kcal% fat	Research Diets	Cat#D12492i
1X Krebs–Ringer Hepes	Alfa Aesar	Cat# J67795
DMEM high glucose media	HyClone	Cat# SH30243.01
Fetal Bovine Serum	ATCC	Cat#30-2020
TRIzol reagent	Fisher Scientific	Cat#15-596-018
SYBR Green Super mix	Bio-Rad	Cat#1725275
4,6-diamidino-2-phenylindole (DAPI)		

Table S1. Patient Data (Related to Figure 8)

Subject	BMI	Gender	SVF preparation?	Time of surgery
1	42.48	M	yes	morning
2	42.71	M	yes	afternoon
3	48.18	M		morning
4	35.24	F		morning
5	35.99	F		afternoon
6	36.3	M	yes	morning
7	36.57	M	yes	morning
8	44.4	F	yes	afternoon
9	43.9	M	yes	morning
10	38.1	F	yes	morning
11	45.73	F		morning
12	39.13	M	yes	morning

Supplementary Table 2

**Oligonucleotides**		
Mouse *18S*<br><br>Fw: CGCCGCTAGAGGTGAAATTC<br><br>Rv: CGAACCTCCGACTTTCGTTCT		
Mouse *Bmal1*<br><br>Fw: GCAGTGCCACTGACTACCAAGA<br><br>Rv: TCCTGGACATTGCATTGCAT		
Mouse *Clock*<br><br>Fw: GGTGGAAGAAGATGACAAGGAC<br><br>Rv: TGTCCATCTTTCTCGCGTTAC		
Mouse *Dbp*<br><br>Fw: AATGACCTTTGAACCTGATCCCGCT<br><br>Rv: GCTCCAGTACTTCTCATCCTTCTGT		
Mouse *Per2*<br><br>Fw: CGCCTAGAATCCCTCCTGAGA<br><br>Rv: CCACCGGCCTGTAGGATCT		
Mouse *Rev-erbα*<br><br>Fw: GGGCACAAGCAACATTACCA<br><br>Rv: CACGTCCCCACACACCTTAC		
Mouse *Rev-erbβ*<br><br>Fw: TCATGAGGATGAACAGGAACC<br><br>Rv: GAATTCGGCCAAATCGAAC		

Supplementary Table 2

Mouse *Ccnd1*<br>Fw: TGCTACCGCACAACGCA<br>Rv: TCAATCTGTTCCTGGCAGGC		
Mouse *Tgf3β*<br>Fw: GGTTACTATGCCAACTTCTG<br>Rv: CACATAGTACAAGATGGTCAG		
Mouse *Ki67*<br>Fw: GCCTTGTGAAGCAGAAGAGAAAAC<br>Rv: CTGGACCTCAAACACCTAACATCAG		[47]
Mouse *Ccna2*<br>Fw: TAAGTACCTGCCTTCACTCATT<br>Rv: TCCAGTCTGTTGTGCCAAT		
Mouse *Pdgfrα*<br>Fw: CAAACCCTGAGACCACAATG<br>Rv: TCCCCCAACAGTAACCCAAG		
Mouse *Cdca8*<br>Fw: CAAGGGACAAAGCATCAGGT<br>Rv: CTCCTCTCCTCCATCCACTG		
Mouse *Ccnb1*<br>Fw: GGAAATTCTTGACAACGGTG<br>Rv: TGCCTTTGTCACGGCCTTAG		

Supplementary Table 2

Mouse *Cenpf*<br><br>Fw: AGTTTGAATCGCTCGTGCTG<br><br>Rv: CGCTTAAGTTCCTGTTCCAGTT		
Mouse *Dcn*<br><br>Fw: TTCCTACTCGGCTGTGAGTC<br><br>Rv: AAGTTGAATGGCAGAACGC		
Mouse *Cd34*<br><br>Fw: GCAGTAAGACCACACCAGCC<br><br>Rv: CAGTATGCCAGTTGGGGAAGT		
Mouse *CD31*<br><br>Fw: AGCCAACAGCCATTACGGTTA<br><br>Rv: TCGACCTTCCGGATCTCACT		
Mouse *CD45*<br><br>Fw: AGCACAACAGAGAATGCCCTT<br><br>Rv: CGTGGATAACACACCTGGATGA		
Mouse *Lep*<br><br>Fw: CTATCCAGAAAGTCCAGGATGACAC<br><br>Rv: CTGGTGAGGACCTGTTGATAGACT		
Mouse *Adipoq*<br><br>Fw: TGTTCCTCTTAATCCTGCCCA<br><br>Rv: CCAACCTGCACAAGTTCCCTT		

Supplementary Table 2

Mouse *Lpl*<br><br>Fw: CGCTCCATTCATCTCTTCATT<br><br>Rv: GGCAGAGCCCTTTCTCAAAGG		
Mouse *PparG*<br><br>Fw: CAAGAATACCAAAGTGCGATCAA<br><br>Fv: GAGCTGGGTCTTTTCAGAATAATAAG		
Human *18S*<br><br>Fw: TGACTCTAGATAACCTCGGG<br><br>Rv: GACTCATTCCAATTACAGGG		
Human *TGF3β*<br><br>Fw: GGTTTTCCGCTTCAATGTGT<br><br>Rv: TATAGCGCTGTTTGGCAATG		
Human *CCND1*<br><br>Fw: AAGGCGGAGGAGACCTGCGCG<br><br>Rv: ATCGTGCGGCATTGCGGC		
